# Analysis of the first genetic engineering attribution challenge

**DOI:** 10.1038/s41467-022-35032-8

**Published:** 2022-11-30

**Authors:** Oliver M. Crook, Kelsey Lane Warmbrod, Greg Lipstein, Christine Chung, Christopher W. Bakerlee, T. Greg McKelvey, Shelly R. Holland, Jacob L. Swett, Kevin M. Esvelt, Ethan C. Alley, William J. Bradshaw

**Affiliations:** 1 Oxford Protein Informatics Group, Department of Statistics, University of Oxford, Oxford, UK; 2 Johns Hopkins Center for Health Security, Johns Hopkins Bloomberg School of Public Health, Baltimore, MD USA; 3 Institute of Public Health Genetics, University of Washington, Seattle, WA USA; 4 DrivenData Inc, Denver, CO USA; 5 altLabs Inc, Berkeley, CA USA; 6 Media Laboratory, Massachusetts Institute of Technology, Cambridge, MA USA

**Keywords:** Machine learning, Computational models, Synthetic biology

## Abstract

The ability to identify the designer of engineered biological sequences—termed genetic engineering attribution (GEA)—would help ensure due credit for biotechnological innovation, while holding designers accountable to the communities they affect. Here, we present the results of the first Genetic Engineering Attribution Challenge, a public data-science competition to advance GEA techniques. Top-scoring teams dramatically outperformed previous models at identifying the true lab-of-origin of engineered plasmid sequences, including an increase in top-1 and top-10 accuracy of 10 percentage points. A simple ensemble of prizewinning models further increased performance. New metrics, designed to assess a model’s ability to confidently exclude candidate labs, also showed major improvements, especially for the ensemble. Most winning teams adopted CNN-based machine-learning approaches; however, one team achieved very high accuracy with an extremely fast neural-network-free approach. Future work, including future competitions, should further explore a wide diversity of approaches for bringing GEA technology into practical use.

## Abstract (web summary)

Identifying the designers of engineered biological sequences would help promote biotechnological innovation while holding designers accountable. Here the authors present the winners of a 2020 data-science competition which improved on previous attempts to attribute plasmid sequences.

## Introduction

Genetic engineering is becoming increasingly powerful, widespread, and accessible, enabling ever-more people to manipulate organisms in increasingly sophisticated ways. As biotechnology advances and spreads, the ability to attribute genetically engineered organisms to their designers becomes increasingly important—both as a means to ensure due recognition and prevent plagiarism, and as a means of holding these designers accountable to the communities their work affects^1–4^. While many academic researchers openly claim credit for their strains and sequences, the provenance of other products—including unpublished work, the products of industrial and government labs, and the work of amateur enthusiasts—is often more difficult to establish.

**Fig. 1:**
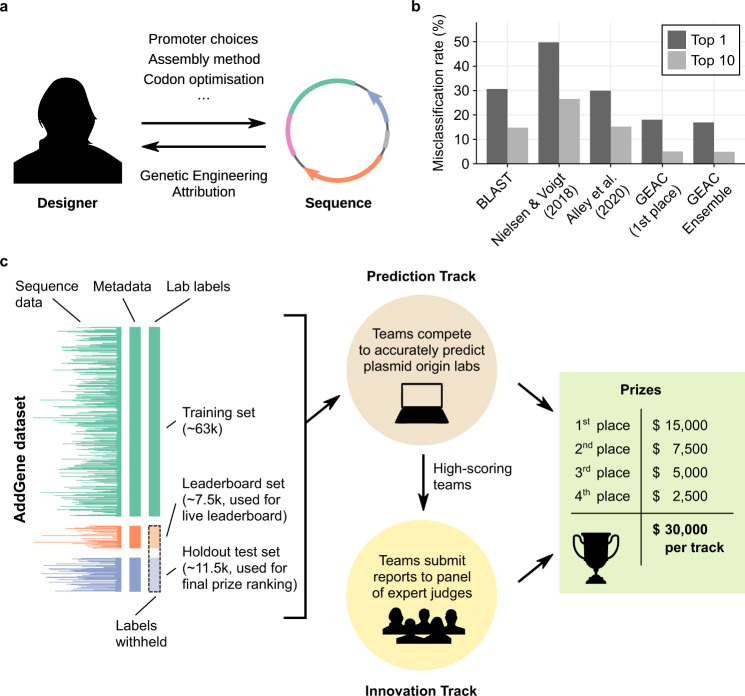
The Genetic Engineering Attribution Challenge. **a** The creation of any synthetic nucleic-acid sequence involves numerous design decisions, each of which leaves a mark in the resulting sequence. Genetic engineering attribution (GEA) aims to use these marks to identify the designer. **b** Misclassification rate (1-(Top-N accuracy)) of past ML approaches to GEA on the Addgene plasmid database, compared to BLAST (left) and the results of the Genetic Engineering Attribution Challenge (GEAC, right). Lower misclassification rates indicate higher accuracy. Our BLAST method achieves higher accuracy than previous implementations; see Methods for details. **c** In the GEAC, teams were provided with engineered plasmid sequences from Addgene, alongside basic metadata for each plasmid. Lab-of-origin labels were provided for the training dataset, but withheld from the leaderboard and holdout test datasets. In the Prediction Track, teams competed to identify these withheld labs-of-origin with the greatest top-10 accuracy. In the Innovation Track, high-scoring teams from the Prediction Track were then invited to submit reports describing their approaches to a panel of expert judges for assessment.

While tools for attributing these products of biotechnology—for *genetic engineering attribution* (GEA)—have historically lagged behind the pace of scientific development, recent years have seen rapid progress^1,2,5,6^. Genetic engineers face many design choices when creating an engineered nucleic-acid sequence, and the sum of these choices constitutes a design signature which, in at least some cases, is detectable by GEA algorithms^2,5^ (Fig. 1a). The more reliably and precisely these algorithms can identify the true designer of a sequence, the greater the potential benefits for accountability and innovation.

Past work on GEA^2,5,6^ has largely focused on predicting the origin lab of plasmid sequences from the Addgene data repository. Performance on this problem has improved rapidly (Fig. 1b). Most recently, Alley et al. used a Recurrent Neural Network (RNN) approach to achieve an accuracy of 70% and a top-10 accuracy (the frequency with which the true lab-of-origin is within the model’s top-10 predictions) of 85%^2^.

A recent publication using a non-machine-learning (ML) pan-genome method reported comparable results, with 76% accuracy (henceforth, “top-1 accuracy”) and 85% top-10 accuracy^6^.

Inspired by these results and the success of past citizen science initiatives^7–10^, we took a community-led approach to the problem, running the first Genetic Engineering Attribution Challenge (GEAC, Fig. 1c) in July-November 2020 (Methods). This public data-science competition, hosted on the DrivenData online platform^11^, consisted of two sequential tracks, termed the Prediction Track and the Innovation Track. In the Prediction Track, teams competed to predict the lab-of-origin of plasmid sequences with the highest possible top-10 accuracy. High-scoring teams from the Prediction Track were then invited to participate in the Innovation Track, writing short reports on their approaches which were assessed by a multidisciplinary panel of expert judges. A prize pool of $30,000 was offered for each track (Supplementary Table Supplementary Table 1).

We focus here on the results of the Prediction Track, which received more submissions and is more amenable to quantitative analysis. The dataset for the Prediction Track was derived from the Addgene dataset used by Alley et al.^2^, comprising sequences and minimal metadata from 81,833 plasmids (Methods). These plasmids were deposited by 3751 origin labs; labs with fewer than ten plasmids were pooled into an auxiliary category (labelled “Unknown Engineered”), leaving a total of 1314 categories for classification. The dataset was divided into training, leaderboard, and holdout test sets (Fig. 1c), with top-10 accuracy on the holdout set determining the final ranking.

## Results

### Core competition outcomes

**Fig. 2:**
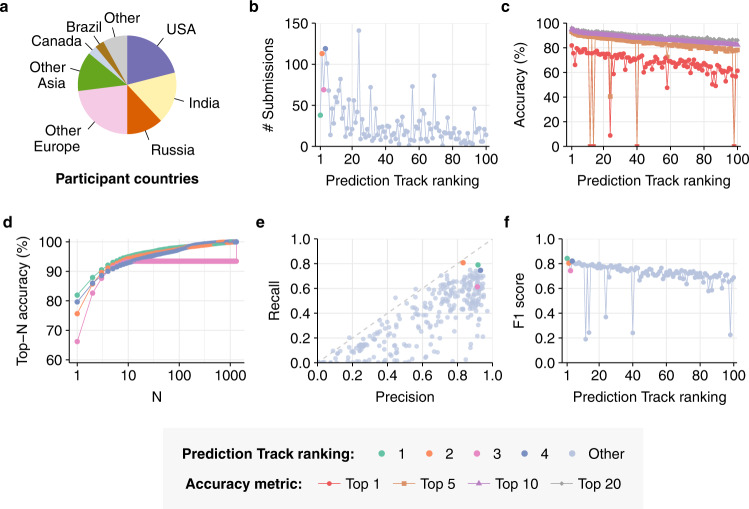
Key Competition Results. **a** Countries of residence of registered competition participants. **b** Total number of submissions made by the top 100 Prediction Track teams. **c** Top-1, −5, −10 and −20 accuracy achieved by each of the top 100 Prediction Track teams. Top-10 accuracy (purple) was used to determine overall ranking and prizes. In all cases, top-5 accuracy is equal to or greater than top-1 accuracy. **d** Top-N accuracy curves of the four prize-winning submissions to the Prediction Track, as a function of N. (**e**) Precision and recall of all 299 Prediction Track teams. Dashed grey line indicates *x* *=* *y*. **f** Macro-averaged F1 score achieved by each of the top 100 Prediction Track teams.

Over 1200 users, from 78 countries (Fig. 2a, Supplementary Table Supplementary Table 2 and Supplementary Table 3), registered to participate in the competition. Of these, 318 users, organised into 299 teams, made at least one submission. There was a strong positive correlation between the number of submissions made by a team and their final top-10 accuracy (Spearman’s *ρ* = 0.82, Fig. 2b, Supplementary Fig. 1): the mean number of submissions made by the top 10% of teams was 49.1, compared to 8.8 for the bottom 90% of teams and 1.4 for the bottom 10%.

The accuracies achieved by Prediction Track teams far exceeded previous work (Fig. 2c, d, Supplementary Figs. 2–3 and 22). 75 teams (25%) achieved higher top-10 accuracies than any previous ML-based GEA model^2,5^; the top-10 accuracy of the highest-scoring team (94.9%) exceeded the previous published record by over 10 percentage points. The other three prizewinning teams also achieved very high prediction accuracy, with top-10 accuracies ranging from 93.0% to 94.4%—all of which exceed the previous record by at least 8 percentage points.

While a single, simple scoring metric was required for the competition, top-10 accuracy represents only one perspective on the performance of an attribution model. To investigate whether the gains seen in this metric represent robust improvements in performance, we broadened our analysis to include top-N accuracy for different values of N (Fig. 2c, d, Supplementary Figs. 2–4). The best models from the competition outperformed previous work across a wide range of *N*-values—in the case of top-1 accuracy, for example, 40 teams (13.4%) outperformed the published record, with the top-scoring team’s accuracy (81.9%) exceeding it by over 11 percentage points. A similar degree of outperformance was observed for top-5 and top-20 accuracy (Supplementary Fig. 3).

In addition to improved accuracy, the best models from the Prediction Track also outperformed previous work on other measures of model performance. The first-, second-, and fourth-place teams all exhibited higher precision and recall than the best previous model, and all four prizewinning teams outperformed the previous best F1 score (Fig. 2e, f, Supplementary Figs. 5–7). As with previous GEA models, most submissions exhibited higher precision than recall, indicating that they returned a higher rate of false negatives than false positives. This tendency can be counterbalanced by looking at a larger number of top predictions from each model—that is, by measuring top-N accuracy for N > 1.

### Evaluating negative attribution with rank metrics

In many important practical applications of GEA, the ability to confidently *exclude* a potential designer (so-called “negative attribution”) can be highly valuable, even if the true designer cannot be identified with confidence^4^. In these contexts, a longer list of candidates presented with very high confidence may be more useful than a shorter list presented with lower confidence.

**Fig. 3:**
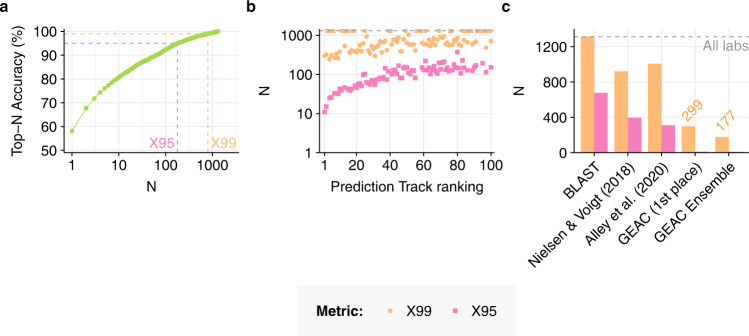
Rank metrics for efficient genetic forensics. **a** For any given lab-of-origin predictor, the X99 score is the smallest positive integer N such that the top-N accuracy of the predictor is at least 99%. Analogous metrics can be defined for other thresholds; for example, the X95 score is the smallest N such that top-N accuracy exceeds 95%. **b** X99 & X95 scores achieved by each of the top 100 Prediction Track teams, on a logarithmic scale. **c** X99 & X95 scores achieved by past ML-based approaches to GEA on the Addgene plasmid database, compared to BLAST (left) and the results of the Genetic Engineering Attribution Challenge (GEAC, right). X99 results for the GEAC 1st place and ensemble models are annotated in orange. Dashed grey horizontal line in (**b**–**c**) indicates the total number of labs in the dataset, which represents the largest possible value of any X-metric on this dataset.

To investigate the degree to which Prediction Track models enable this sort of confident negative attribution, we developed a new metric. The *X99 score* of a predictor is the minimum positive integer N such that the top-N accuracy of that predictor is at least 99% (Fig. 3a). Analogous metrics can be defined for other accuracy thresholds; for example, the X95 score of a predictor is the smallest value of N such that its top-N accuracy is at least 95%. The lower the values of these two metrics, the better the predictor is able to confidently focus subsequent investigation on a manageable set of candidates.

**Fig. 4:**
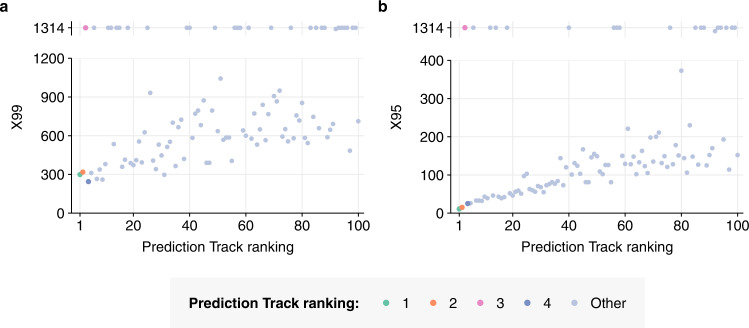
X-metrics in detail. **a** X99 and (**b**) X95 scores achieved by each of the top 100 Prediction Track teams, on separate linear scales. In each case, outlier values are shown in a separate panel above. There are 1314 lab categories in the dataset.

We computed X99 and X95 scores for every team in the Prediction Track, as well as for previously published GEA models (Fig. 3b, c, Fig. 4, Supplementary Figs. 8–13 and 22). The lowest X99 score achieved by any previous model on the same dataset was 898 (using the CNN model of Nielsen & Voigt 2018), while the lowest previous X95 score was 311 (using the RNN model of Alley et al. 2020). In contrast, the lowest X99 score achieved in the Prediction Track was 244, achieved by the fourth-place Prediction Track team—a 73% reduction compared to the previous record. The X99 score of the first-place team was 299. The lowest X95 score achieved in the Prediction was 11, achieved by the first-place team—a 96% reduction. The competition results thus represent a dramatic improvement in negative attribution capability.

### Improving performance with ensembling

Ensembles of multiple models routinely improve performance across a wide range of ML problems^12–14^. Indeed, all prizewinning teams in the Prediction Track made use of some sort of ensemble to generate their predictions (see below). We therefore hypothesised that further ensembling could achieve even greater performance.

Our simple ensemble of the winning models (Methods) achieved marginally higher top-10 accuracy than the 1st-place team, showing a gain of 0.2 percentage points (95.1 vs 94.9%, Fig. 1b, Supplementary Figs. 3 and 14). The improvement seen in top-1 accuracy was larger, with an increase of 1.4 percentage points (83.1% vs 81.9%). This degree of top-1 accuracy approaches the best top-10 accuracies previously reported in the literature^2,6^. The ensemble model also achieved the highest F1 score of any ML-based GEA model to date (Supplementary Fig. 6), reflecting a better balance between precision and recall than was achieved by individual winning teams.

By far the largest improvement from the ensemble was seen in the X99 negative-attribution metric discussed above (Fig. 3c, Supplementary Fig. 9). The ensemble achieved an X99 score of 177, compared to 299 for the overall competition winner and 244 for the team with the lowest X99 (a 27.5% reduction). This dramatic improvement suggests that significant further gains in X99 may be possible, further increasing the practical applicability of GEA models.

### Effects of large composite classes on prediction accuracy

As discussed above, small labs in the competition dataset were pooled into a single auxiliary category, labelled “Unknown Engineered”. This category was the largest in the dataset, making up 7.5% of sequences, compared to 2.4% for the largest unique lab (Supplementary Fig. 15). Given this frequency, it is possible that teams could inflate their Prediction Track scores by always including Unknown Engineered in their top 10 lab-of-origin guesses. Indeed, high-scoring teams included Unknown Engineered in their top-10 guesses at a rate far exceeding its true frequency, and the frequency with which they did so was correlated with their overall top-10 accuracy (Spearman’s *ρ* = 0.57, Supplementary Fig. 16a, b).

As a result, the top-10 accuracy achieved by most teams on Unknown Engineered sequences far exceeded that of sequences assigned to a unique lab category, inflating teams’ top-10 accuracy overall (Supplementary Fig. 16c). Previous GEA models exhibited similar behaviour (Supplementary Fig. 17). In general, however, the effect was marginal: for the top 10% of teams, the average top-10 accuracy on unique (non-Unknown-Engineered) labs was only 0.7 percentage points lower than their accuracy on the entire dataset. Nevertheless, these results illustrate an important weakness in this approach to handling small and unseen labs in GEA datasets.

### Calibration of competition models

Deep-learning models are often overconfident in their predictions^15^. This can cause problems for their interpretation, especially in cases, like GEA, where the evidence from such models needs to be weighed alongside multiple other data sources. Under these circumstances, it is useful to measure the calibration of model predictions, and potentially to take steps to improve that calibration prior to use^15–17^.

Under conventional definitions of calibration, a predictor is considered to be well-calibrated if events it predicts with probability *Y* occur 100 × *Y* % of the time. Common metrics for measuring calibration in this vein include the Expected Calibration Error (ECE) and Maximum Calibration Error (MCE)^15^, which measure the average and maximum absolute deviation observed across some number of binned ranges (Methods).

Previous work on GEA has included calibration analysis. Alley et al.^2^ found that their RNN-based model was reasonably well-calibrated (ECE = 4.7%, MCE = 8.9%); our reanalysis of that model’s predictions returned similar values (ECE = 5.9%, MCE = 8.9%, Supplementary Fig. 18). We also found that this RNN model was far better calibrated than other previous attempts at GEA, especially with regard to MCE (Supplementary Fig. 18). Given these results, we decided to investigate the calibration of Prediction Track teams.

The MCEs and ECEs exhibited by Prediction Track teams varied widely, and were only modestly correlated with Prediction Track ranking (Spearman’s *ρ* vs ECE = 0.15, *ρ* vs MCE = 0.38, Supplementary Fig. 19). Among the prizewinning teams, the 4th-place winner performed best in terms of calibration, achieving results comparable to Alley et al. (ECE = 3.4% and MCE = 11.8%, Supplementary Fig. 18). The other prizewinners exhibited worse performance, with an average ECE of 23.5% and an average MCE of 27.7%. This reflects generally poor calibration among teams generally: the top 10% of teams achieved an average ECE of 17.5% and an average MCE of 33.5% (Supplementary Fig. 20).

These results are not surprising: it is common for deep-learning models to be very miscalibrated^15^, and models in the Prediction Track were not penalised for poor calibration. Nevertheless, our results demonstrate that the relative rankings produced by these models are generally more informative than their specific probability estimates.

### Strategies used by prize-winning teams

At the close of the competition, the prizewinning teams shared their model code with organisers, allowing us to investigate the strategies they employed^18,19^. At a high level, the 1st-, 2nd- and 4th-place teams took remarkably similar approaches, with all of them employing ensembles containing at least one convolutional network^12,13,20^. However, the precise structure of these ensembles, including the number and size of the component networks^14^ and the preprocessing methods employed, varied considerably. Several teams normalised or augmented their dataset using the reverse complement of each sequence, and one team used principal component analysis^21^ on BLAST features as input to their neural network. The 1st-place team combined multiple CNNs with a model based on *k*-mer counts, which appeared to complement the CNNs. Unlike the previous best-performing GEA model^2^, none of the winning teams employed an RNN-based approach.

In sharp contrast to other winning teams, the 3rd-place Prediction Track team did not employ neural networks at all. Instead, they took a radically different approach, using *k-*mer kernels, naive Bayes^21^, soft masks and rank merging^22^. In addition to achieving top-10 accuracy comparable with the best neural-network-based solutions, this approach was also dramatically faster: 0.66 CPU hours to train and run, compared to >40 GPU hours for similarly performant deep-learning-based solutions—a 1000-fold difference in the cost of compute (Methods, Supplementary Table 4). This approach had substantially worse top-1 accuracy (Fig. 2d) and X95/X99 scores (Fig. 4) than the other winning solutions; however, these shortfalls may result from over-optimisation for the top-10-accuracy metric used in the competition, rather than inherent limitations.

## Discussion

By most quantitative metrics we investigated, the first GEAC was a resounding success. Along its core evaluation metric, top-10 accuracy, winning teams achieved dramatically better results than any previous attempt at GEA, with the top-scoring team and all-winners ensemble both beating the previous state-of-the-art by over 10 percentage points. Similarly large gains were seen for the more-conventional top-1-accuracy metric, despite submissions receiving no additional benefit from placing the true lab in first place.

To investigate whether models at this level of performance might be useful in practice, we developed two new metrics: X95 and X99. These metrics evaluate whether a model can generate a manageable list of candidates while reliably (with 95 or 99% confidence) including the true lab-of-origin. At the 95% level, the best models from the competition essentially solved this problem for the Addgene dataset, reducing X95 from over 300 to <15. Progress on X99 was similarly dramatic: our ensemble of the winning models achieved an X99 score of 177, an 80% reduction compared to previous work. Nevertheless, at the 99% level, further progress is needed before the problem can be considered solved.

While high-scoring competition teams performed very well on accuracy and X95/X99 metrics, not all the metrics we investigated showed such positive results. In particular, winning models were much less well-calibrated than some previously published models, making it difficult to take the specific probabilities of their predictions at face value. Recall and F1 scores also showed further room for improvement. These suboptimal results are not surprising: ECE, MCE, recall, and F1 all focus on the single top prediction made by a model for each sequence, but models in the competition were rewarded for ranking the true lab anywhere in their top 10 predictions. Future models, trained under broader optimisation incentives, will hopefully achieve similar or greater accuracy while excelling along a wider variety of metrics; further focus on X99 in particular could help reward models that are more robustly useful.

While the results of this competition are highly encouraging, it is important to keep in mind the gulf between the form of attribution problem presented here, and the problems to which GEA might be applied in practice. In many respects, the Addgene dataset—a large, well-curated database of broadly similar plasmid sequences, with the authorship of each sequence made freely available—represents a highly simplified form of GEA. While the availability of this dataset has been critical to the development of GEA approaches to date, if they are to be practically useful, attribution models will eventually need to generalise far beyond this initial scenario.

From this perspective of practical application, the fact that so many teams outperformed the previous best models in this field is promising, as it suggests that a wide variety of approaches could perform well on this problem. That one of the prizewinning teams adopted a very fast and completely neural-network-free approach to the problem is also encouraging, since speed of deployment and ease of retraining will be important in many applications of attribution technology. Future exploration of these and other desirable properties, alongside improvements in accuracy, will be an important part of bringing GEA into regular use. Further work on model interpretability will also be key, to enable human experts to incorporate GEA results alongside other forms of evidence.

At the same time, we envisage that investigating a wider range of methods, such as equivariant neural networks^23^, transformers/attention methods^24^ and uncertainty-aware approaches^25–28^ may prove fruitful. Alternative approaches to handling small and unseen classes in GEA datasets—such as data augmentation^29,30^, anomaly detection^31–33^, or the use of more robust evaluation metrics^34,35^—should also be explored. Given the rapid improvement in GEA models to date, and the gains made during this competition, we are optimistic that further dramatic improvements, even to the point of practical application, may be within sight.

## Methods

### Competition design

#### Overview

The GEAC was a free, online, public data-science competition held on the DrivenData competition platform^11^. The competition was organised and sponsored by altLabs, Inc in collaboration with DrivenData, Inc. The competition was open to all individuals over the age of 18, from any country, with the exception of (i) officers, directors, employees and advisory board members of altLabs or DrivenData, (ii) immediate family members and housemates of those individuals, and (iii) individuals who are residents of countries designated by the United States Treasury’s Office of Foreign Assets Control.

As discussed in the main text, the competition consisted of two sequential tracks: the Prediction Track and the Innovation Track, each of which is described in detail below. The Prediction Track ran from August 18 to October 19, 2020, while the Innovation Track ran from October 20 to November 1, 2020. Results for both tracks were announced on January 26, 2021. Both tracks had a total prize pool of US$30,000; the distribution of prize money among winning teams is specified in Supplementary Table 1. All prize money was provided by altLabs, Inc.

#### The Prediction Track

In the Prediction Track, participants attempted to guess the lab-of-origin of plasmid sequences from the Alley et al. dataset (see below). Participants were given access to both training data and labels from the training set, while labels from the leaderboard and holdout test sets were withheld. The top-10 accuracy of each submission on the leaderboard set was immediately reported to the submitting team upon submission, and the best top-10 accuracy scores on this set for each team were continuously displayed on a public leaderboard during the competition. The top-10 accuracy of each submission on the holdout test set was not reported until after the Prediction Track had closed, and was used to determine the final competition ranking. Prizes were awarded to the four teams who achieved the highest top-10 accuracy scores on this private test set.

#### The Innovation Track

Following closure of the Prediction Track, teams that achieved a top-10 accuracy of at least 75.6% were invited to participate in the Innovation Track. This threshold was based on an earlier estimate of BLAST top-10 accuracy (see below). To compete in this track, participants were asked to submit short reports (maximum 4 pages, maximum 2 figures), which were then reviewed by a team of judges (see below). describing how their approach would contribute to solving real-world attribution problems. Prizes were awarded to teams who exhibited novel and creative approaches to the problem, or who demonstrated that their algorithms possessed useful properties other than raw accuracy. The full text of the Innovation Track problem description is available in the Supplementary Note.

Submitted reports were assessed by a team of 12 judges, including experts in synthetic biology, bioinformatics, biosecurity, and machine learning. Each judge reviewed a group of six submissions; assignment of submissions into these groups was performed randomly, with the constraints that each possible pair of submissions must be reviewed by at least two judges and that each individual submission must be reviewed by the same number of judges.

To avoid issues arising from differences in scoring practices between judges, each judge was asked to rank the submissions they received, with a rank of 1 indicating the best submission. Prizes were awarded to the four teams who achieved the smallest average rank across judges. In the event of a two-way tie, the process was repeated using only those judges who reviewed both submissions; this was sufficient to obtain four unique prizewinners in this case.

### Data preparation

Data for the GEAC was provided by Alley et al.^2^, and comprised all plasmids deposited in the Addgene repository up to July 27th 2018—a total of 81,834 entries. For each plasmid, the dataset included a DNA sequence, along with metadata on growth strain, growth temperature, copy number, host species, bacterial resistance markers, and other selectable markers. Each of these categorical metadata fields was re-encoded as a series of one-hot feature groups:

- **Growth strain:** growth_strain_ccdb_survival, growth_strain_dh10b, growth_strain_dh5alpha, growth_strain_neb_stable, growth_strain_other, growth_strain_stbl3,growth_strain_top10, growth_strain_xl1_blue
- **Growth temperature:** growth_temp_30, growth_temp_37, growth_temp_other
- **Copy number:** copy_number_high_copy, copy_number_low_copy, copy_number_unknown
- **Host species:** species_budding_yeast, species_fly, species_human, species_mouse,species_mustard_weed, species_nematode, species_other, species_rat, species_synthetic,species_zebrafish
- **Bacterial resistance:** bacterial_resistance_ampicillin, bacterial_resistance_chloramphenicol, bacterial_resistance_kanamycin, bacterial_resistance_other, bacterial_resistance_spectinomycin
- **Other selectable markers:** selectable_markers_blasticidin, selectable_markers_his3, selectable_markers_hygromycin, selectable_markers_leu2, selectable_markers_neomycin, selectable_markers_other,selectable_markers_puromycin, selectable_markers_trp1, selectable_markers_ura3, selectable_markers_zeocin

In addition to the sequence and the above metadata fields, the raw dataset also contained unique sequence IDs, as well as separate IDs designating the origin lab. For the competition, both sequence and lab IDs were obfuscated through 1:1 replacement with random alphanumeric strings.

The number of plasmids deposited in the dataset by each lab was highly heterogeneous (Supplementary Fig. 21). Many labs only deposited one or a few sequences—too few to adequately train a model to uniquely identify that lab. To deal with this problem, Alley et al. grouped labs with fewer than 10 data points into a single auxiliary category labelled “Unknown Engineered”. This reduced the number of categories from 3751 (the number of labs) to 1314 (1313 unique labs + Unknown Engineered).

In addition to issues with small labs, the dataset also contains “lineages” of plasmids: sequences that were derived by modifying other sequences in the dataset. This could potentially bias accuracy measures by introducing dependencies between entries in the training and test sets. To deal with this issue, Alley et al. inferred lineage networks among plasmids in the dataset, based on information in the complete Addgene database acknowledging sequence contributions from other entries. More specifically, lineages were identified by searching for connected components within the network of entry-to-entry acknowledgements in the Addgene database (see Alley et al.^2^ for more details).

The data were partitioned into train, validation, and test sets, with the constraints that (i) every category have at least three data points in the test set, and (ii) all plasmids in a given lineage be assigned to a single dataset. Following the split, the training set contained 63,017 entries (77.0%); the validation set contained 7466 entries (9.1%); and the test set contained 11,351 entries (13.9%).

For the GEAC, these three data partitions were reassigned based on the needs of the competition: the training set was provided to the participants for model development, including the true (though obfuscated, see above) lab IDs. The validation and test sets, meanwhile, were repurposed as the leaderboard and holdout test sets of the competition. One entry with a 1nt sequence was dropped from the leaderboard set, leaving a total of 7465 entries.

The test and leaderboard sets were shuffled together, and provided to participants without the accompanying lab IDs; as described above, participants’ top-10 accuracy on the validation set was used to determine their position in the public leaderboard during the competition, while their top-10 accuracy on the test set was used to determine the final ranking and prizewinners. To avoid overfitting, participants were not shown their results on the holdout test set until the end of the competition, at which point participants were ranked based on the top-10 accuracy of their most recent submission on that test set.

### Data integrity

In order to minimise competitor access to Addgene data during the GEAC, a number of steps were undertaken during the design and execution of the competition, including:

- The source of the data was not disclosed to participants;
- Plasmid and lab IDs were obfuscated in the competition dataset, raising the barrier to potential cheating;
- In order to receive any prize money, high-scoring participants had to submit their model code to DrivenData for independent verification—including visual inspection for obvious cheating, validation of performance on the test dataset, and verification on a separate dataset of Addgene sequences collected after the competition.

### Computing the BLAST benchmark

Previous implementations of GEA using BLAST^36^ have reported top-1 accuracies of just over 65% and top-10 accuracies of roughly 75%^2^. During the preparation of this manuscript, we found that a small modification of this attribution algorithm (specifically, replacing use of the quicksort algorithm^37^ with mergesort^38^) resulted in equal top-1 accuracy, while substantially increasing top-N accuracy for N > 1 (Supplementary Fig. 3). We have used the results from this modified algorithm in the main text, while presenting both sets of results side-by-side in the supplementary material. Under our implementation, the procedure followed by both algorithms can be summarised as follows:

- Sequences from the training set were extracted into a FASTA file, then used to generate a BLAST nucleotide database.
- Sequences from the test set were extracted into a FASTA file, then aligned to the training-set database, with an *E*-value threshold of 10.
- Alignments reported by BLAST were sorted in ascending order of *E*-value. The original implementation used quicksort for this sorting step, while our modified algorithm used mergesort. (In the latter but not the former case, this is equivalent to sorting in descending order of bit score.)
- The lab IDs corresponding to each training-set sequence were identified, and the sorting results were filtered to include only the first result for each lab-ID/test-set-sequence combination. The remaining hits for each test-set sequence were ranked in ascending order of occurrence in the dataset.
- Finally, top-N accuracy was calculated as the proportion of test-set sequences for which the ID of the true origin lab was assigned a rank less than or equal to N.

BLAST version 2.10.1 was used to generate the baseline.

For the purpose of calculating calibration (Supplementary Fig. 18), these ranks were reversed (so that the best match had the highest rank) and normalised using softmax.

### Other baselines

Predictions on the competition test set for deteRNNt^2^ and a reproduction of the CNN model developed by ref. [5] were provided by ref. [2]. Top-N accuracy, X-metrics, calibration indices, and other metrics were re-computed from scratch based on these files.

### Post-competition analysis

Demographic information on the competition was collected using Google Analytics (Universal Analytics). Other data were analysed using python 3.7 and R version 4.1. Figures were plotted using ggplot2 version 3.3.1.

Each submission to the Prediction Track consisted of a *J* *×* *K* prediction matrix, where *J* is the number of sequences in the holdout test set (11,351) and *K* is the total number of lab classes in that test set (1314). Each entry in this matrix ostensibly reflected a predicted probability of the corresponding lab being the true lab-of-origin for that sequence, with the entries in each row summing to unity.

To compute accuracy metrics for each team for this analysis, we first generated a rank matrix from their prediction matrix. In this matrix, the lab with the highest predicted probability for a given sequence was assigned rank 1, the second-highest prediction rank 2, and so on. To prevent teams achieving high scores by giving uniform predictions for large numbers of labs, tied predictions were assigned the maximum rank. Given this rank matrix, the top-N accuracy for any N could thus be computed as the proportion of rows for which the true lab was assigned a rank of N or less.

Given these accuracy scores, the X99 score could be computed as the minimum positive integer *N* such that top-*N* accuracy is at least 99%. This metric can be generalised to other thresholds, where X*R* is the minimum positive integer *N* such that top-*N* accuracy is at least *R*%. X95, X90 and X80 scores were all computed in this way.

For the purposes of calculating precision and recall, the number of true positives, false positives and false negatives were computed separately for each lab class for each submission. For a given class, the number of true positives ${tp}$ was defined as the number of times in the test set that that class was correctly assigned rank 1 (i.e. assigned rank 1 when it was in fact the true lab-of origin); the number of false positives ${fp}$ as the number of times it was incorrectly assigned rank 1; and the number of false negatives ${fn}$ as the number of times it was incorrectly assigned rank >1. Precision and recall for each class were then calculated as ${tp}/({tp}+{fp})$ and recall as ${tp}/({tp}+{fn})$, and the F1 score for each class as the harmonic mean of its precision and recall. The overall precision and recall for each team were computed as the arithmetic mean of its class-specific precisions and recalls, respectively, while the macro-averaged F1 score was computed as the arithmetic mean of its class-specific F1 scores.

### Calibration

Following Guo et al.^15^ we checked whether predictions had frequentist calibration of their probabilistic forecasts. To estimate the expected accuracy from finite samples, we grouped predictions into 15 interval bins of equal size. We let ${B}_{m}$ be the set of indices of samples whose prediction confidence falls into the intervals $(\frac{m-1}{M},\frac{m}{M}]$. The *accuracy* of bin ${B}_{m}$ is then defined as

1 $${{{{{\rm{acc}}}}}}\left({B}_{M}\right)=\frac{1}{\left|{B}_{m}\right|}\mathop{\sum}\nolimits_{{B}_{m}}1({\hat{{{{{\rm{y}}}}}}}_{i}={y}_{i})$$

where ${\hat{{{{{\rm{ y }}}}}}}_{i}$ and ${y}_{i}$ are the (top-1) predicted and true class labels for sequence $i$ and $\left|{B}_{m}\right|$ is the number of samples in bin ${B}_{m}$. The *average confidence* within bin ${B}_{m}$ is defined as

2 $${{{{{\rm{conf}}}}}}\left({B}_{M}\right)=\frac{1}{\left|{B}_{m}\right|}\mathop{\sum}\nolimits_{{B}_{m}}{\hat{{{{{{\rm{p}}}}}}}}_{i}$$

where ${\hat{{{{{{\rm{p}}}}}}}}_{i}$ is the predicted probability assigned to class ${\hat{{{{{{\rm{y}}}}}}}}_{i}$ for sequence *i*. The expected deviation between confidence and accuracy can then be estimated using the *expected calibration error* (ECE):

3 $${{{{{\rm{ECE}}}}}}={\sum }_{m=1}^{M}\frac{\left|{B}_{m}\right|}{n}\left|{{{{{\rm{acc}}}}}}\left({B}_{M}\right)-{{{{{\rm{conf}}}}}}\left({B}_{M}\right)\right|$$

where $n$ is the total number of samples. The *maximum calibration error* (MCE) estimates the worst-case deviation from the binning procedure as:

4 $${{{{{\rm{MCE}}}}}}=\mathop{{{\max }}}\limits_{{{{{{\rm{m}}}}}}\in \{1,\ldots,M\}}\left|{{{{{\rm{acc}}}}}}\left({B}_{M}\right)-{{{{{\rm{conf}}}}}}\left({B}_{M}\right)\right|$$

### Ensemble

To ensemble the four prizewinning teams from the Prediction Track, the probability assigned to each lab for each plasmid sequence was averaged between the top 4 classes, with equal weight given to each class. That is, the prediction for sequence $i$ to lab $j$ was given by:

5 $${p}_{{ij}}=\frac{1}{4}{\sum }_{k=1}^{4}{p}_{{ijk}}$$

where $k$ indexes over the methods and ${p}_{{ijk}}$ is the prediction score given for sequence $i$ to lab $j$, by method $k$.

### Amazon web server compute costs

Approximate costing for machine learning methods were calculated using Amazon EC2 on-demand pricing. We assumed a single GPU machine with sufficient memory (128 GB) costing $1.14 per hour (g3.8×large). This totals $51.30 for 45 h of GPU time. For the CPU based methods, which required 20GB of solid-state drive, an x2gd.medium instance, costing $0.08 per hour, would be sufficient. This totals $0.05 for the 0.66 CPU hours used.

### Robustness analysis

To assess the robustness of the ranking of the winning teams to choice of validation dataset, the lab-of-origin predictions for the set of sequences were subsampled so that predictions were only retained for 80% of the sequences. Sampling was performed without replacement for each subsample. The rank order of predictions was re-computed on this subsampled dataset, and from here we computed metrics of interest including top-1 accuracy, top-10 accuracy and X99 score.To generate a distribution of scores, this resampling strategy was performed 1000 times. Distributions were compared using the KS-test; all pairwise comparisons between teams on all metrics (top-1 accuracy, top-10 accuracy, and X99) were significantly different at *p* < 0.01.

### Reporting summary

Further information on research design is available in the Nature Portfolio Reporting Summary linked to this article.

## Supplementary information

Supplementary Information

Reporting Summary

## Data Availability

Summarised competition data, including all data files required to generate all figures in this paper, are publicly available online at https://github.com/willbradshaw/geac/^39^. Due to licensing agreements, competition datasets and prediction data are available on request to the corresponding author at wjbrad@mit.edu.

## Code availability

Code related to this paper is publicly available online at https://github.com/willbradshaw/geac/^39^.
