# Supplementary material for: Analysis of the first genetic engineering attribution challenge
Source: Nat Commun. 2022 Nov 30;13:7374. doi: 10.1038/s41467-022-35032-8 (PMC9712580; doi:10.1038/s41467-022-35032-8)
Supplement: Supplementary file 1 — Supplementary Information [file 41467_2022_35032_MOESM1_ESM.pdf]

Analysis of the first Genetic Engineering Attribution Challenge – Supplementary Information

Oliver M. Crook[1], Kelsey Lane Warmbrod[2,3], Greg Lipstein[4], Christine Chung[4], Christopher W. Bakerlee[5], T. Greg McKelvey Jr.[5], Shelly R. Holland[5], Jacob L. Swett[5], Kevin M. Esvelt[5,6], Ethan C. Alley[5,6,*], and William J. Bradshaw[5,6,†]

[1] Oxford Protein Informatics Group, Department of Statistics, University of Oxford, Oxford, United Kingdom
[2] Johns Hopkins Center for Health Security, Johns Hopkins Bloomberg School of Public Health, Baltimore, MD, USA
[3] Institute of Public Health Genetics, University of Washington, Seattle, WA, USA
[4] DrivenData Inc, Denver, CO, USA
[5] altLabs Inc, Berkeley, CA, USA
[6] Media Laboratory, Massachusetts Institute of Technology, Cambridge, MA, USA
[*] For correspondence: ethan@altlabs.tech
[†] For correspondence: wjbrad@mit.edu

1. Supplementary Tables

Supplementary Table 1: Distribution of prizes. The same distribution was used for both the Prediction Track and the Innovation Track. Both tracks had a total prize pool of $30,000, from an overall total of $60,000.

Competition Rank	Prize money ($)
1st	$15,000
2nd	$7,500
3rd	$5,000
4th	$2,500

Supplementary Table 2: Competition engagement. Number of users who viewed, joined, or submitted predictions to the Genetic Engineering Attribution Challenge. Many submitting teams consisted of a single individual, while some included multiple users.

Activity	# Individuals
Visited competition website	8101
Registered as participant	1211
Submitted predictions (users)	318
Submitted predictions (teams)	299

Supplementary Table 3: Geographic origin of competition visitors and participants. Proportion of site visitors and registered participants from each continent and country.

Continent	Country / Region	% site visitors	% registered participants
North America	USA	29	21
	Canada	3	3
	Other N. America	< 1	< 1
South America	Brazil	2	3
	Other S. America	2	1
Asia	India	13	17
	Other Asia[1]	15	13
Europe	Russia	8	12
	UK	4	3
	Netherlands	2	3
	Germany	3	3
	France	2	3
	Other Europe	12	11
Africa	All countries	3	5
Oceania	All countries	2	2

[1] China accounted for 2% of site visitors and ≤2% of participants.

Supplementary Table 4: Runtime and computational costs of winning models. All code was reproduced in Amazon EC2 P3-large instance, which uses V100 cards.

Pred. Track Ranking	CPU/ GPU	# of Processors	Training Time	Testing Time	Disk Memory (GB)	Virtual Memory (GB)	Specialized hardware
1st	GPU / CPU	1 / 8	65 hours	1 hour	200	64	RTX 2080
2nd	GPU	1	1 week	12 hours	200	128	RTX 2080
3rd	CPU	1	40 minutes	10 minutes	30	16	30 GB SSD
4th	GPU	2	8 hours	1 hour	200	128	RTX 2080

2. Supplementary Figures

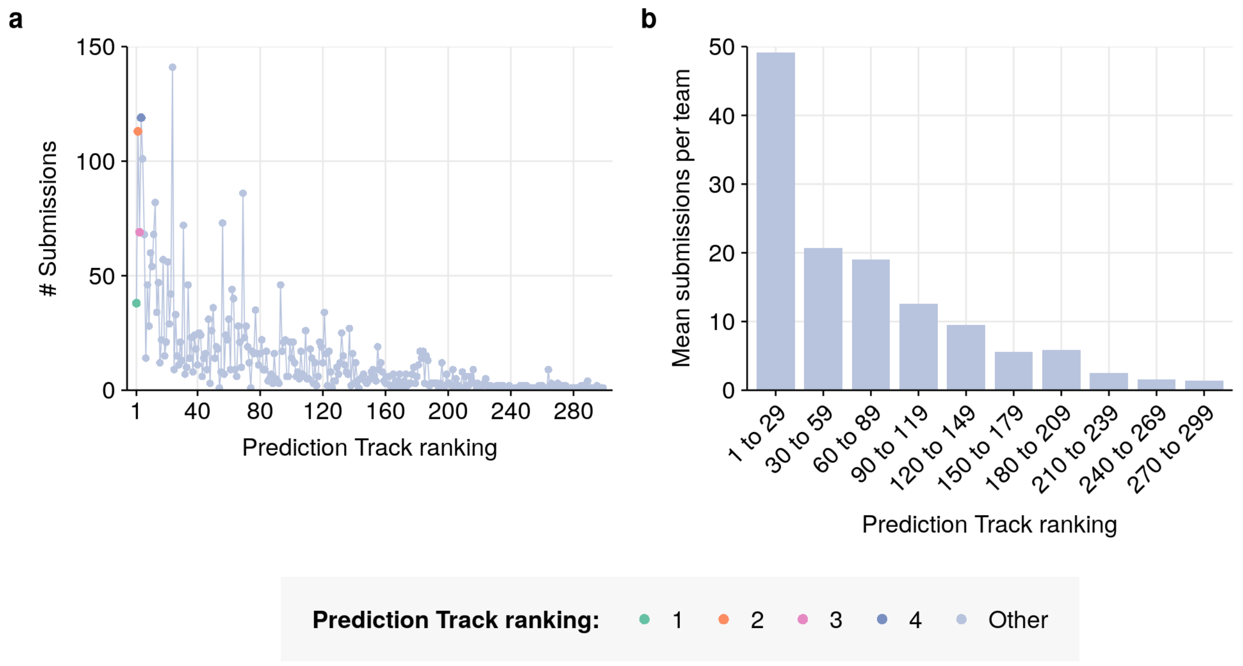

Supplementary Figure 1: Submissions to the Prediction Track. (a) Total number of submissions made by each Prediction Track team. (b) Mean number of submissions of each decile of teams, as ranked by top-10 accuracy.

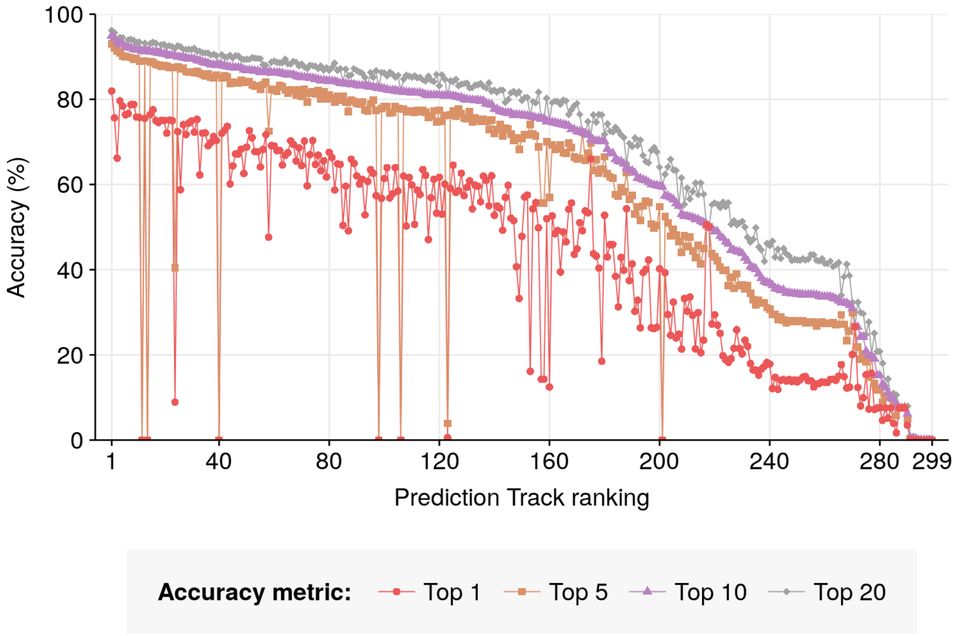

Supplementary Figure 2: Prediction Track accuracy. Top-1, -5, -10 and -20 accuracy achieved by each team in the Prediction Track.

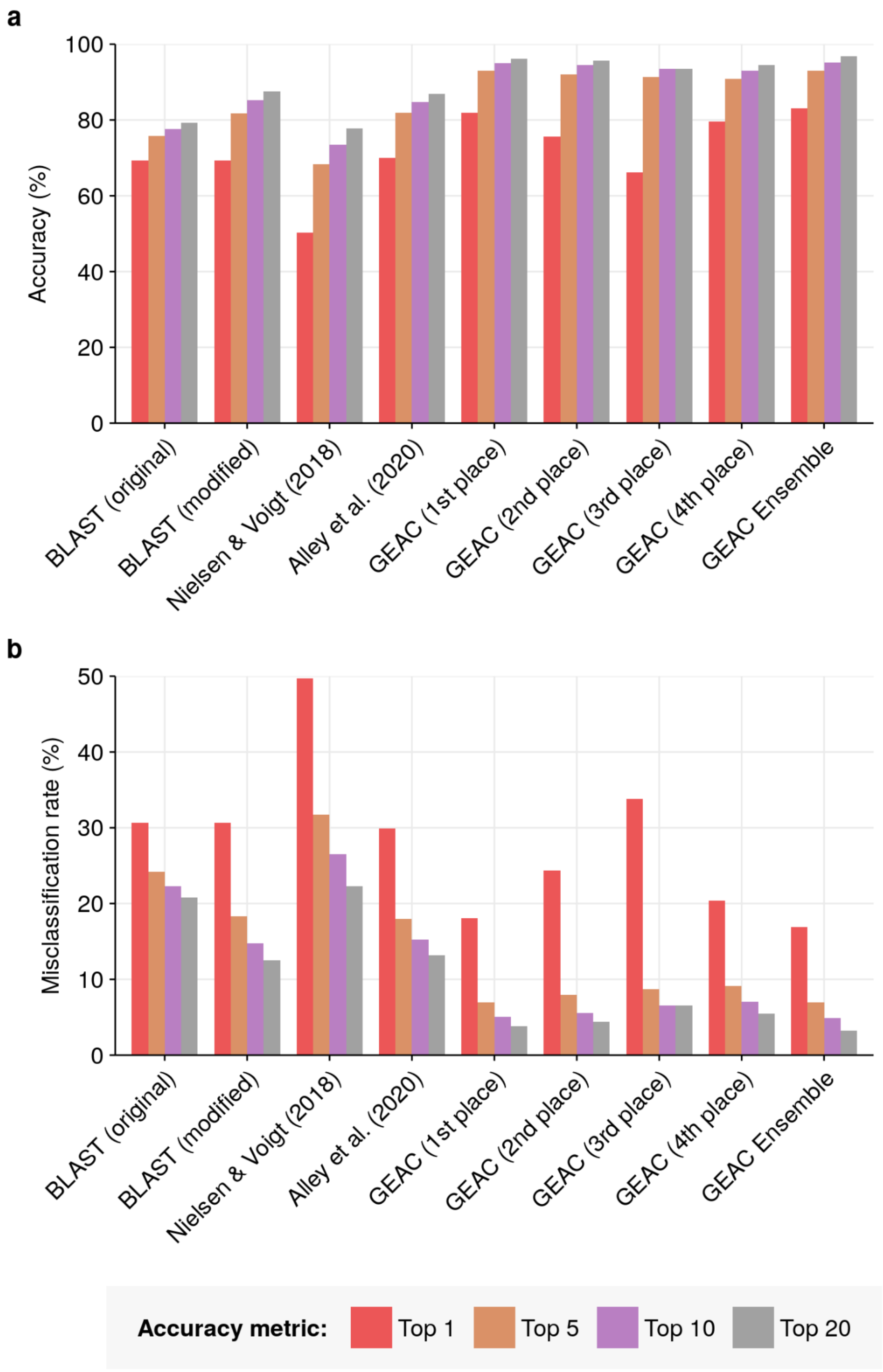

Supplementary Figure 3: Key model accuracy. (a) Top-1, -5, -10 and -20 accuracy achieved by Prediction Track winners and ensemble, as compared to BLAST (see Methods) and previously-published ML-based GEA models. (b) Misclassification rate (1-(Top-N accuracy)) for those models.

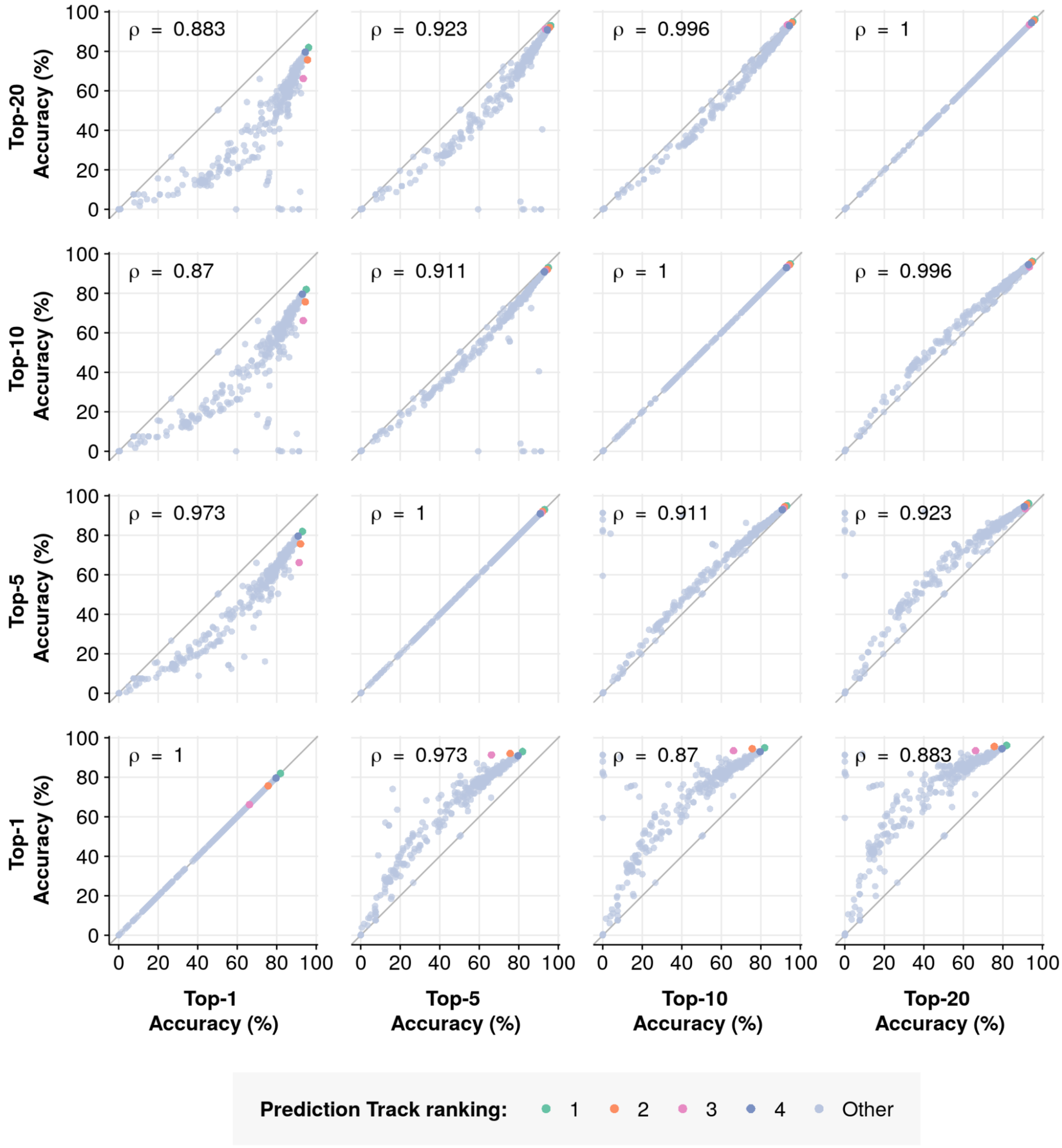

Supplementary Figure 4: Relationship between Prediction Track accuracy metrics. Scatter plots of the Top-1, -5, -10 and -20 accuracies achieved by each Prediction Track team, plotted against one another. Grey lines indicate *x=y*. Text in each panel indicates the Spearman rank correlation coefficient between each pair of metrics.

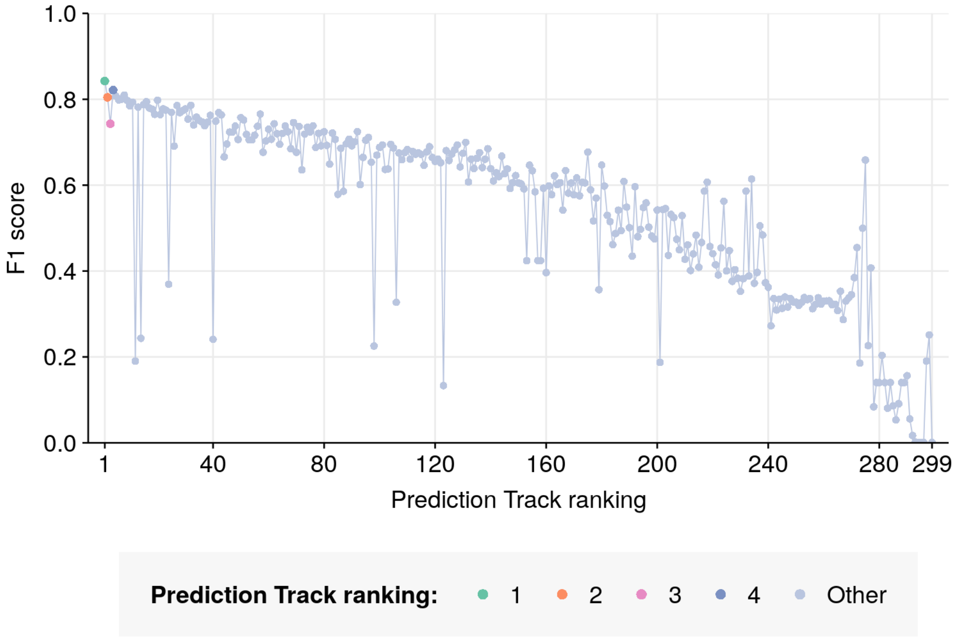

Supplementary Figure 5: Prediction Track F1 scores. Macro-averaged F1 score achieved by each team in the Prediction Track.

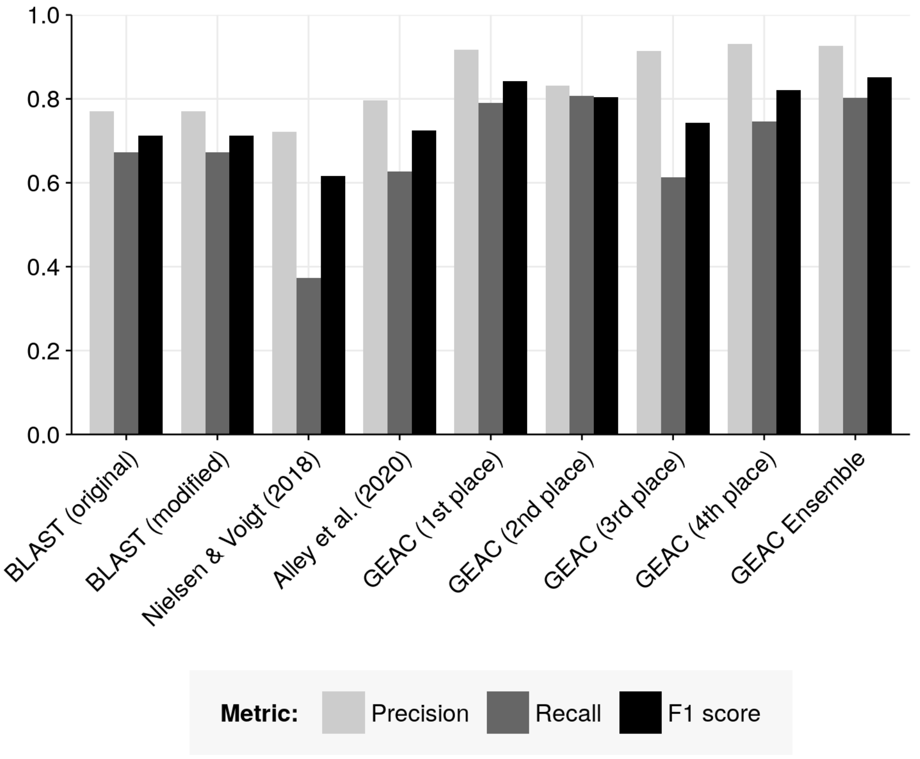

Supplementary Figure 6: Precision, recall, and F1 for key models. Precision, recall, and macro-averaged F1 scores achieved by Prediction Track winners and ensemble, as compared to BLAST and previously-published ML-based GEA models, on a logarithmic scale.

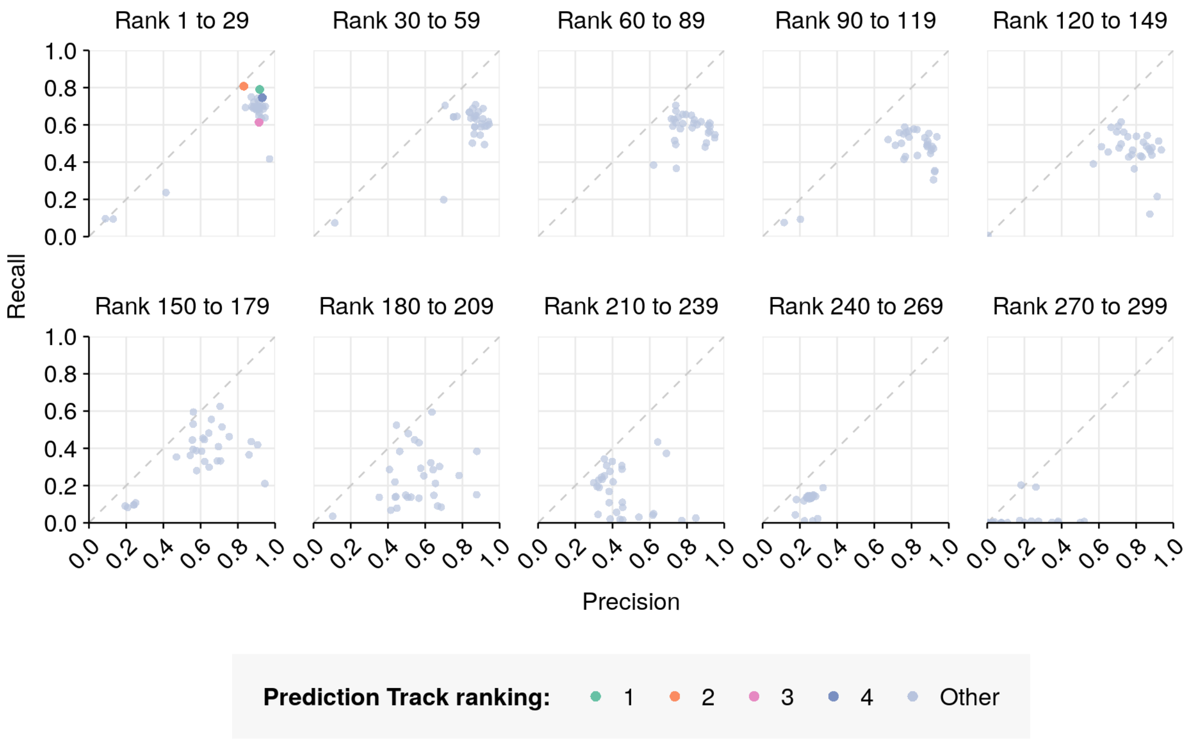

Supplementary Figure 7: Prediction Track precision and recall. Precision and recall achieved by each team in the Prediction Track, separated by score (i.e. top-10 accuracy) decile.

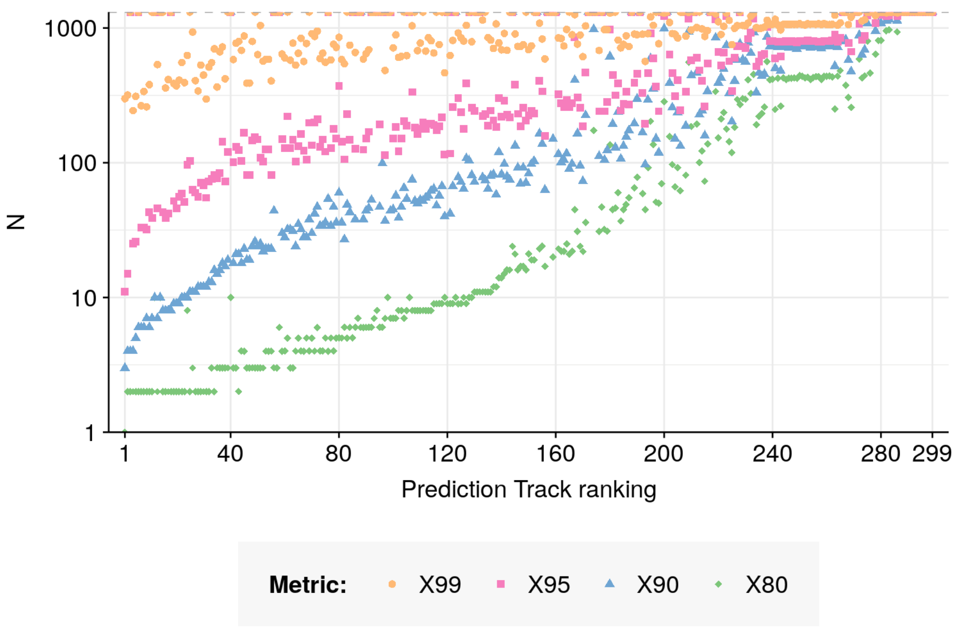

Supplementary Figure 8: X-metrics for Prediction Track teams. X99, X95, X90 and X80 scores achieved by each Prediction Track team, on a logarithmic scale.

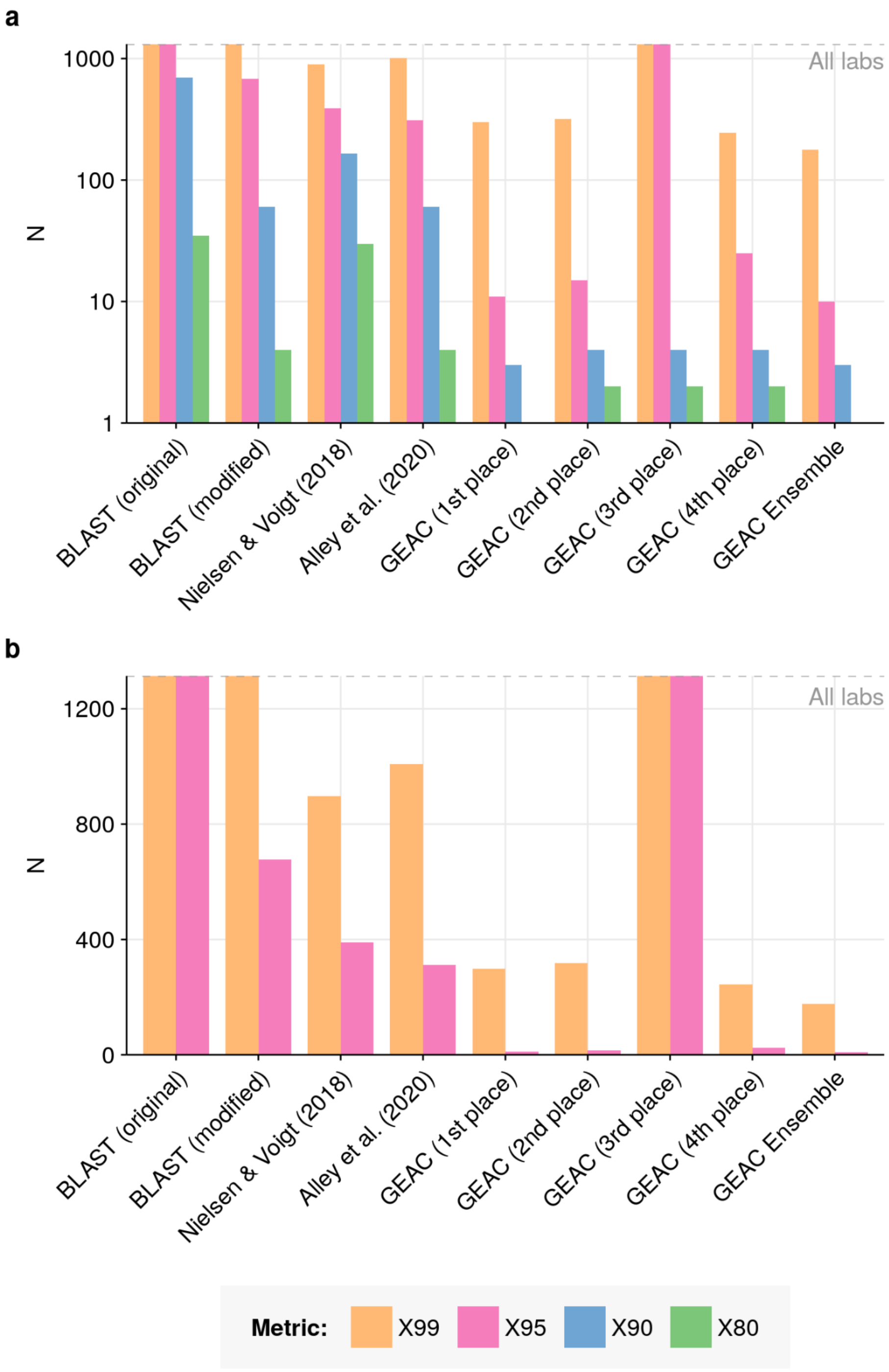

Supplementary Figure 9: X-metrics for key models. (a) X99, X95, X90 and X80 scores achieved by Prediction Track winners and ensemble, as compared to BLAST and previously-published ML-based GEA models, on a logarithmic scale. (b) X99 and X95 scores of those models, on a linear scale. Dashed grey horizontal lines indicate the total number of labs in the dataset, which represents the largest possible value of any X-metric on this dataset.

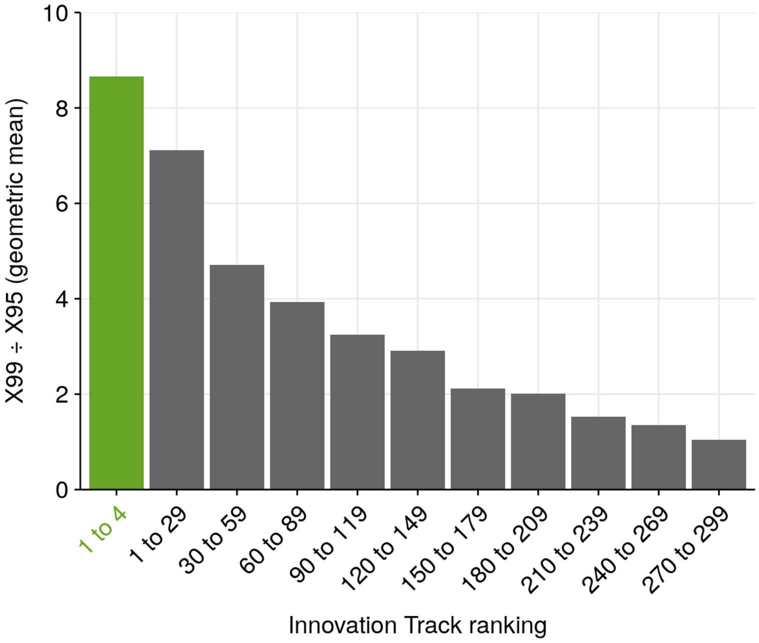

Supplementary Figure 10: X99 vs X95. Geometric mean of the ratio between X99 to X95 scores for teams in each rank decile of the Prediction Track (grey bars) as well as for the four winning teams (green bar).

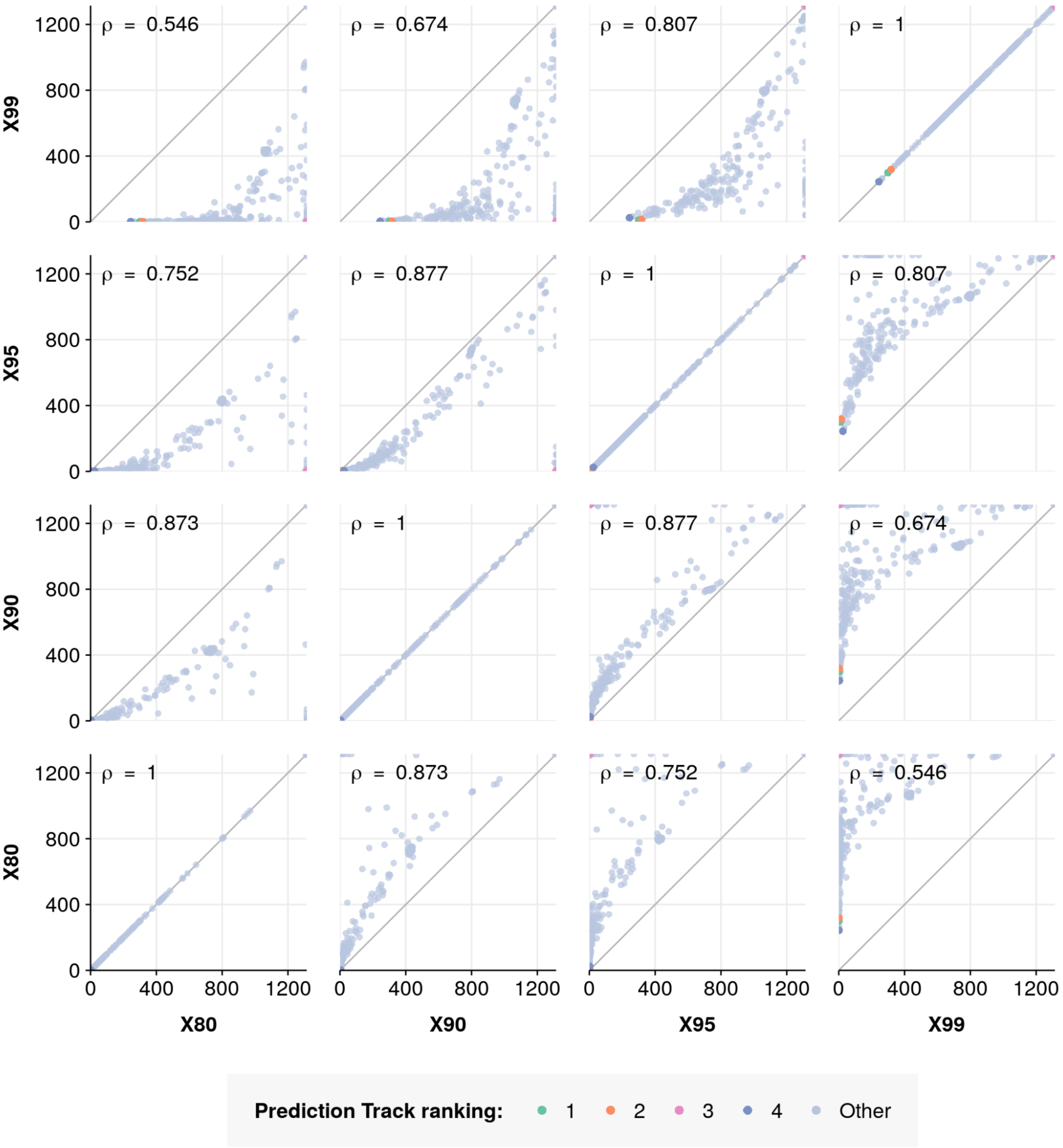

Supplementary Figure 11: Relationship between Prediction Track X-metrics. Scatter plots of the X99, X95, X90, and X80 scores achieved by each Prediction Track team, plotted against one another. Grey lines indicate *x*=*y*. Text in each panel indicates the Spearman rank correlation coefficient between each pair of metrics.

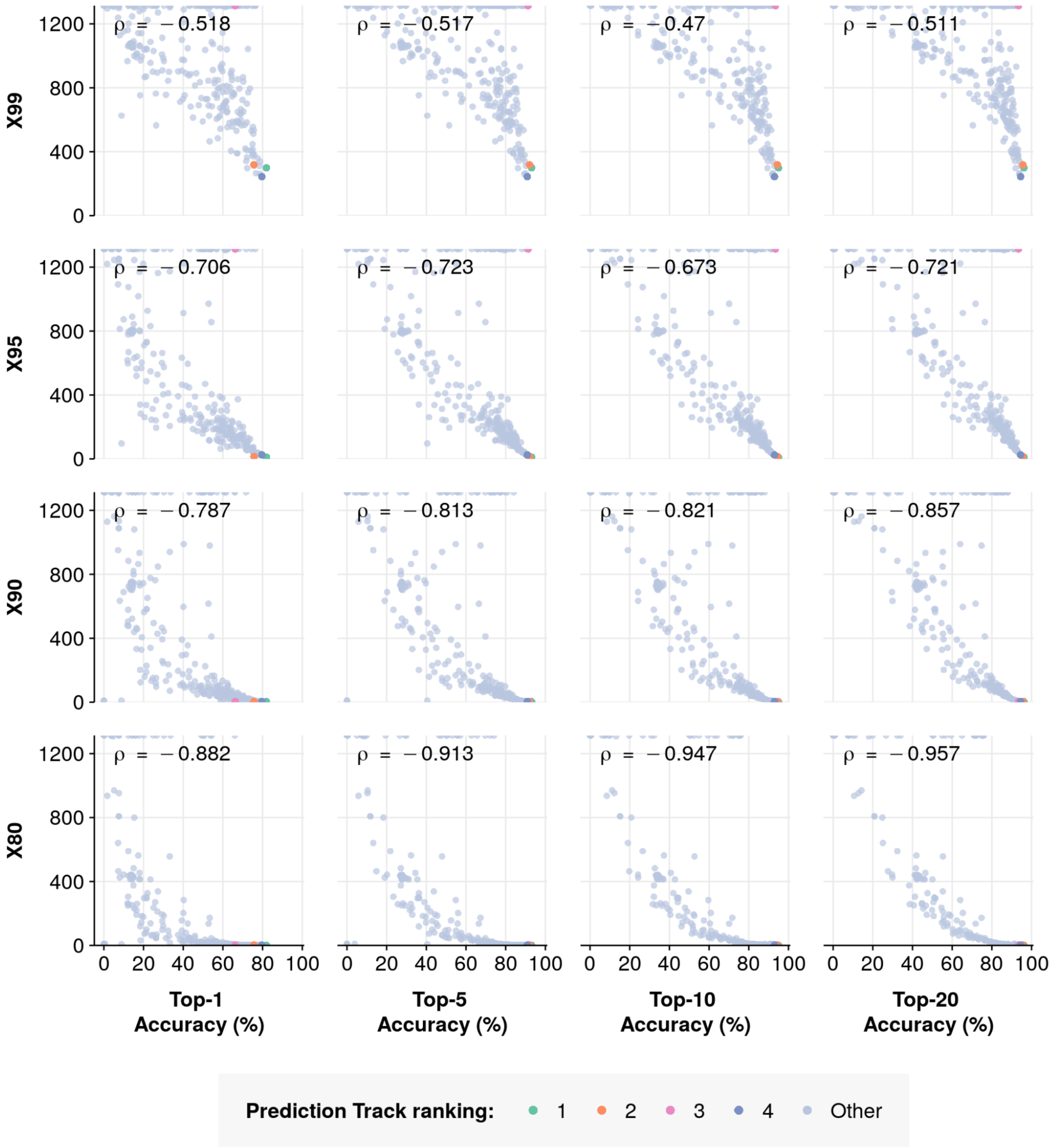

Supplementary Figure 12: Relationship between Prediction Track accuracy and X-metrics (all teams). Scatter plots of the X99, X95, X90, and X80 scores achieved by each Prediction Track team, plotted against their top-1, -5, -10 and -20 accuracies. Text in each panel indicates the Spearman rank correlation coefficient between each pair of metrics.

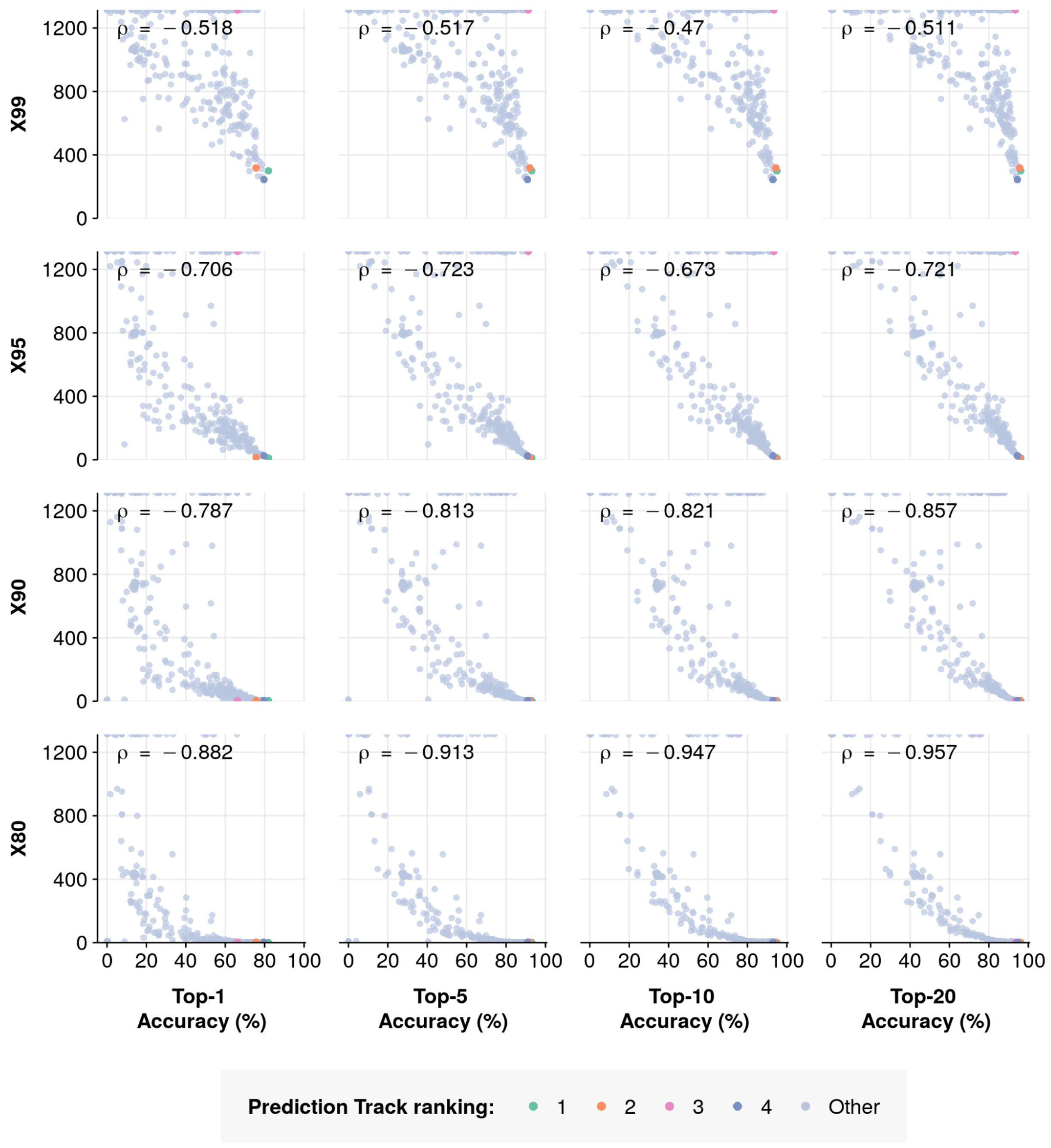

Supplementary Figure 13: Relationship between Prediction Track accuracy and X-metrics (outliers removed). Scatter plots of the X-scores and accuracy metrics achieved by each Prediction Track team, excluding those with X-metrics equal to the number of labs. Many teams only returned positive probabilities for their top 10 candidates for each sequence, preventing them from achieving 95% (or 99%) accuracy without including the entire set of labs as candidates. Text in each panel indicates the Spearman rank correlation coefficient between each pair of metrics, given the aforementioned filtering.

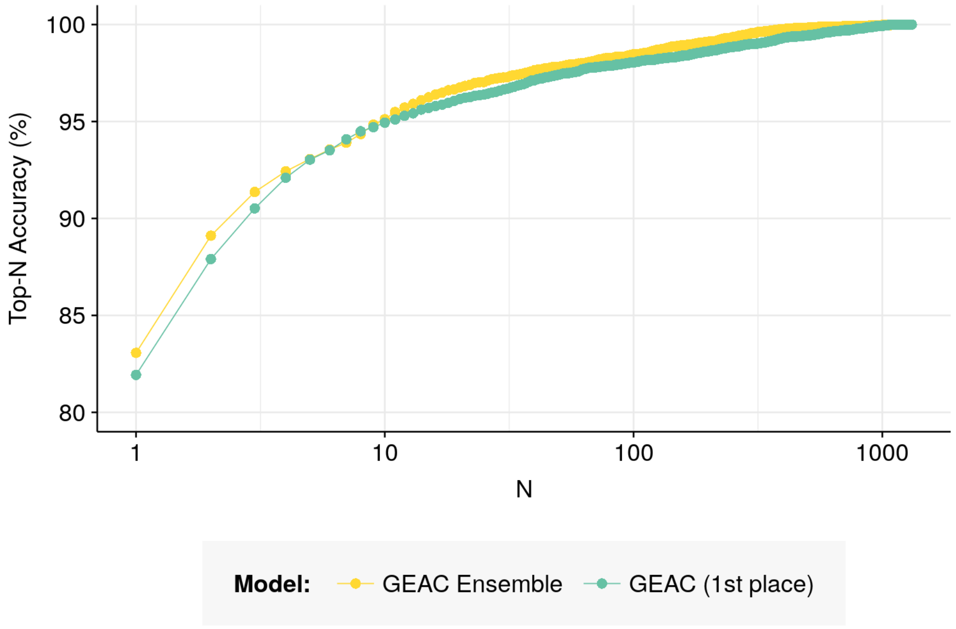

Supplementary Figure 14: Ensemble top-N accuracy. Top-N accuracy vs N for the GEAC ensemble model, as compared to the top-scoring Prediction Track team. For most values of N the Ensemble outperforms the competition winner.

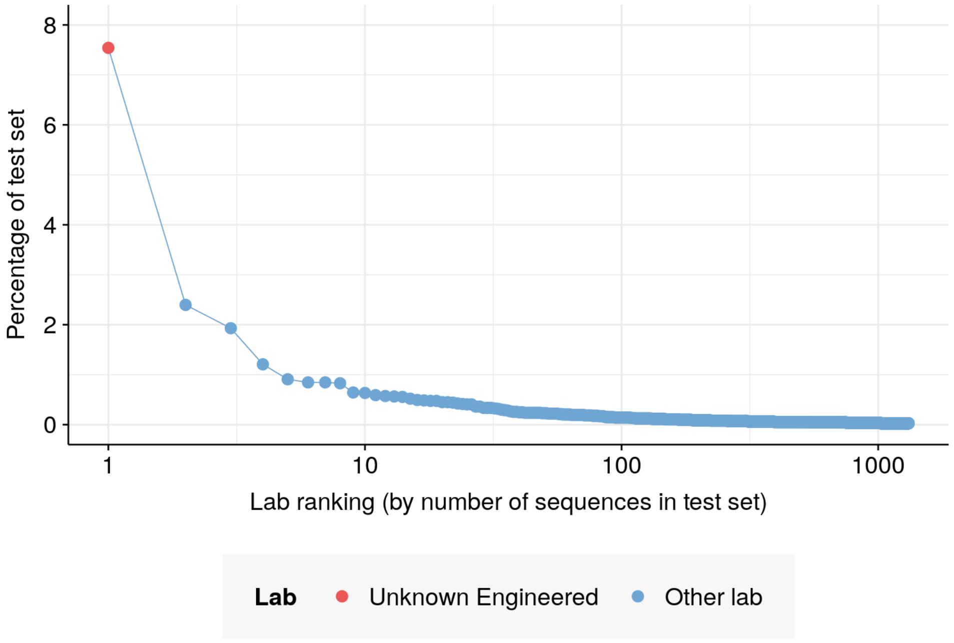

Supplementary Figure 15: Lab composition of the GEAC test set. Percentage of sequences in the competition test set accounted for by each lab category. Blue points indicate unique labs; the red point indicates the “Unknown Engineered” bucket category (Methods). Labs are ordered on the x-axis in descending order of prevalence in the test set.

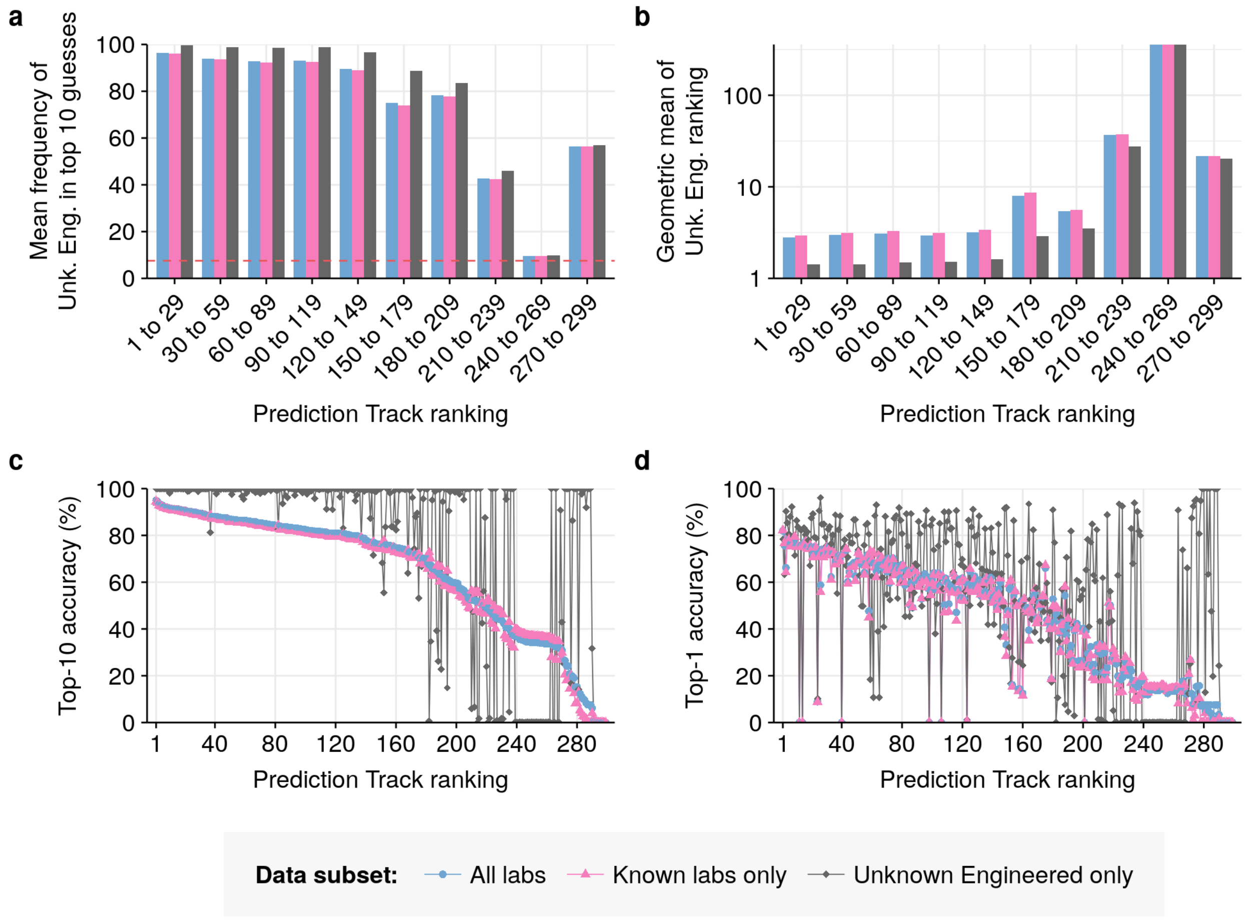

Supplementary Figure 16: The Unknown Engineered category. (a) Mean percentage of sequences in the test set for which submissions in each decile of teams included Unknown Engineered in their top-10 lab-of-origin guesses, compared to the true frequency of the category (7.5%, dashed red line). (b) Geometric mean rank of Unknown Engineered, across all sequences in the test set, for each decile of teams. (c) Top-10 accuracy achieved by each team in the Prediction Track on each of three subsets of the test set: sequences from all lab categories, sequences from known labs only (excluding Unknown Engineered), and Unknown Engineered sequences only. (d) Top-1 accuracies achieved on the same data subsets.

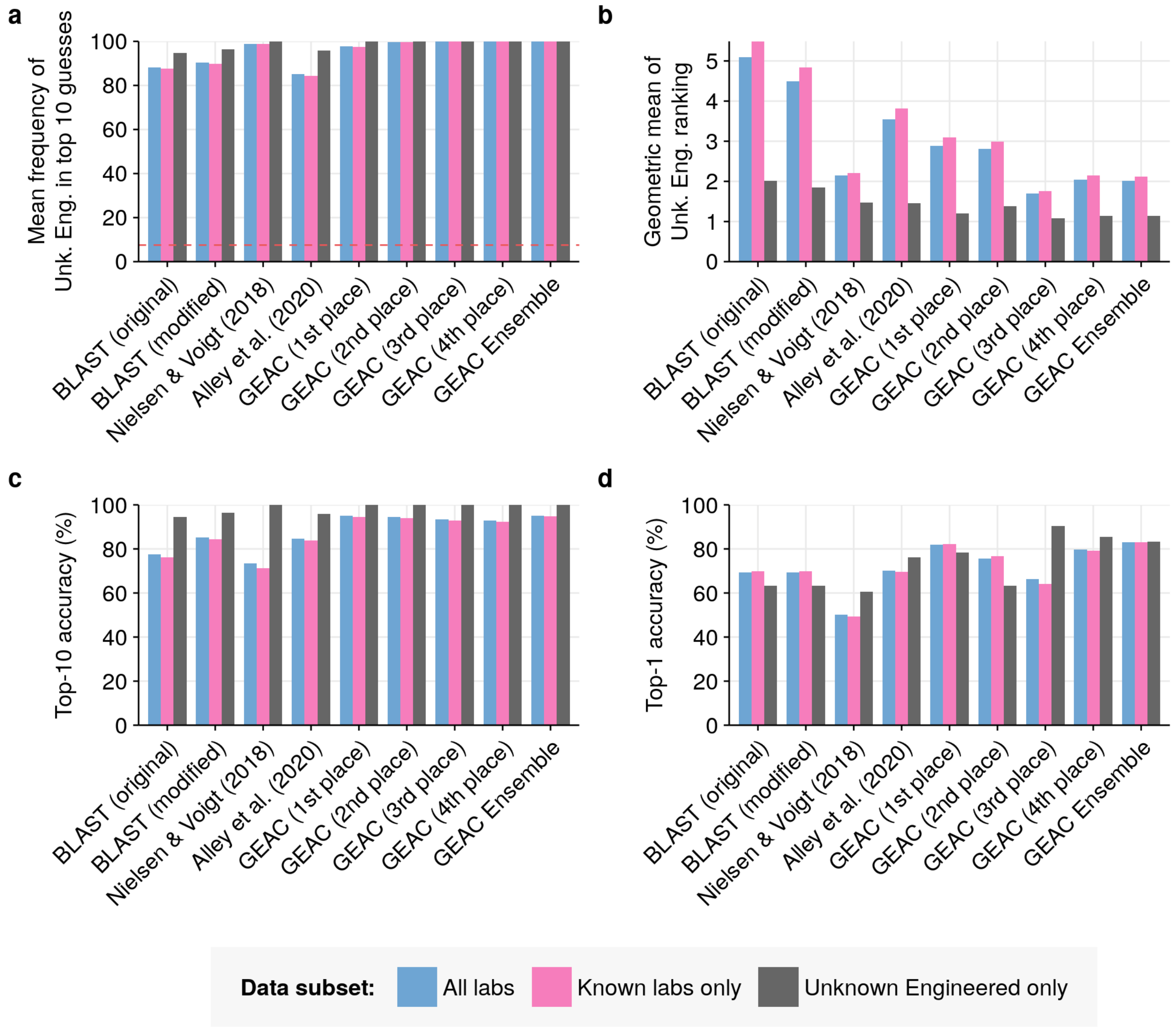

Supplementary Figure 17: Unknown Engineered metrics for key models. (a) Mean percentage of sequences in the test set for which key models (BLAST, past ML GEA models, GEA winners and ensemble) included Unknown Engineered in their top-10 lab-of-origin guesses, compared to the true frequency of the category (7.5%, dashed red line). (b) Geometric mean rank of Unknown Engineered, across all sequences in the test set, for each key model. (c) Top-10 accuracy achieved by each key model on each of three subsets of the test set: sequences from all lab categories, sequences from known labs only (excluding Unknown Engineered), and Unknown Engineered sequences only. (d) Top-1 accuracies achieved on the same data subsets.

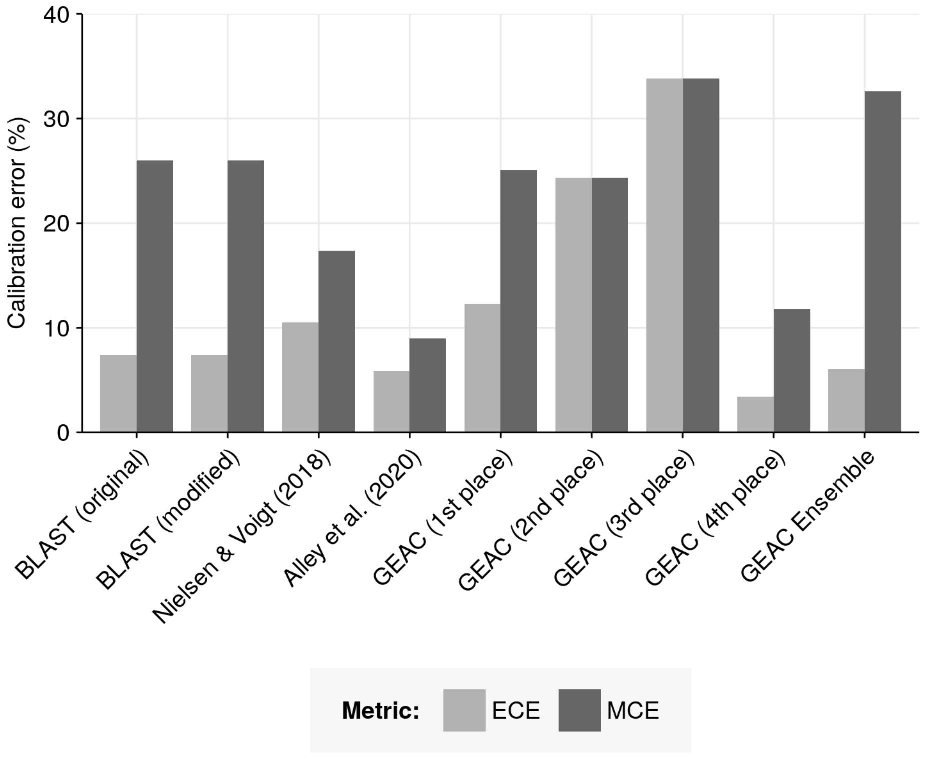

Supplementary Figure 18: Calibration of key models. Expected calibration error (ECE) and maximum calibration error (MCE) of Prediction Track winners and ensemble, as compared to BLAST and previously-published ML-based GEA models.

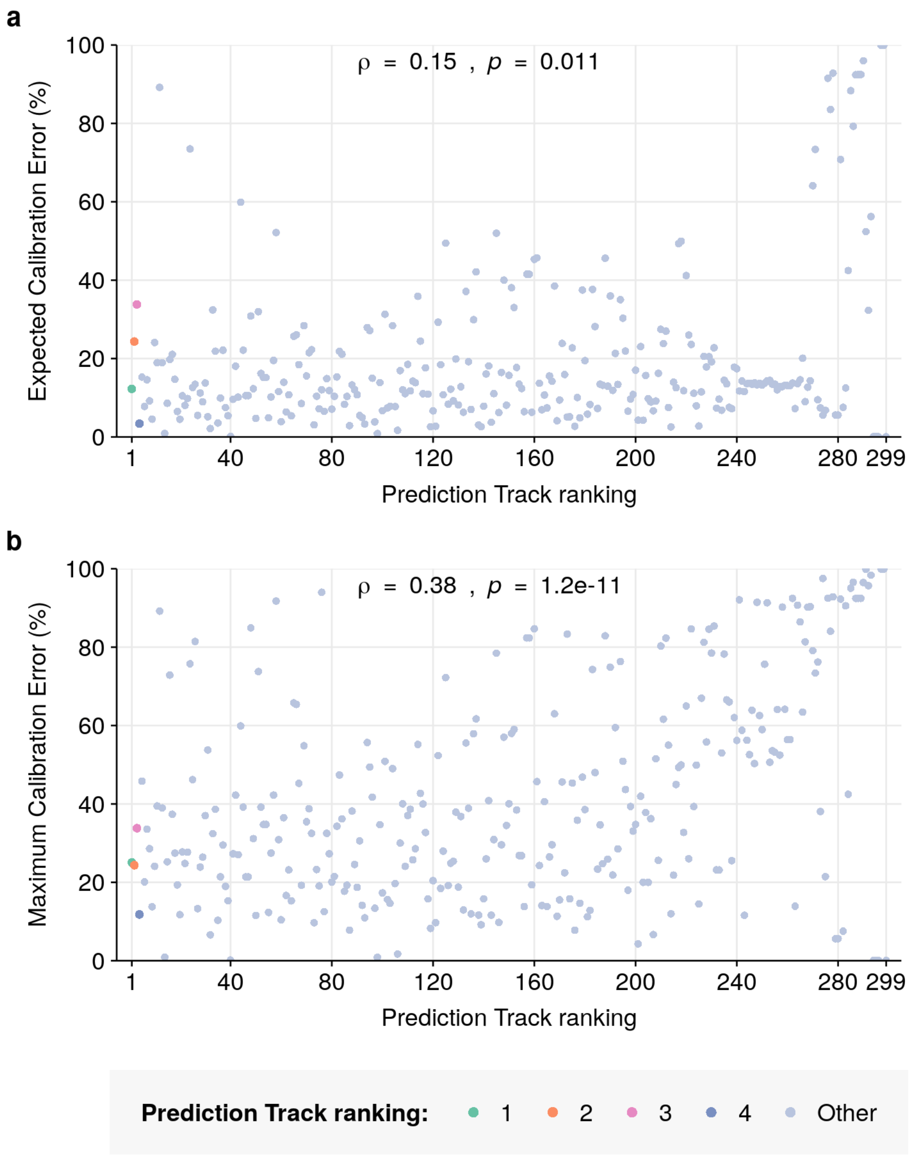

Supplementary Figure 19: Calibration of Prediction Track teams. (a) Expected and (b) maximum calibration error of each team in the Prediction Track. ρ indicates Spearman's Rank correlation value between calibration error metrics and Prediction Track ranking.

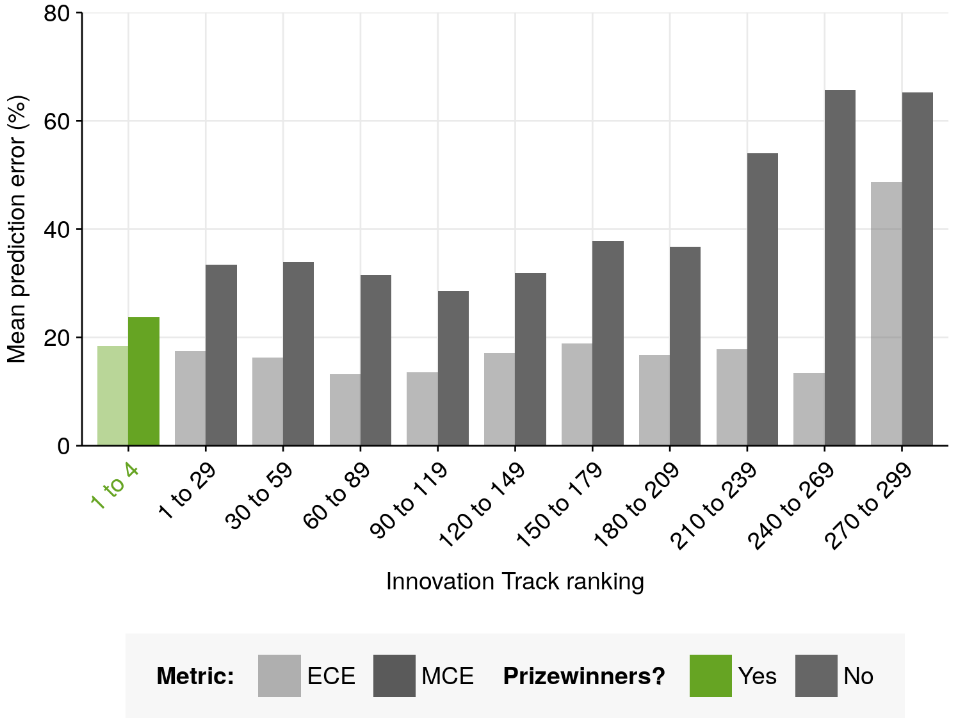

Supplementary Figure 20: Calibration of Prediction Track teams by decile. Expected and maximum calibration errors for teams in each rank decile of the Prediction Track (grey bars) as well as for the four winning teams (green bar).

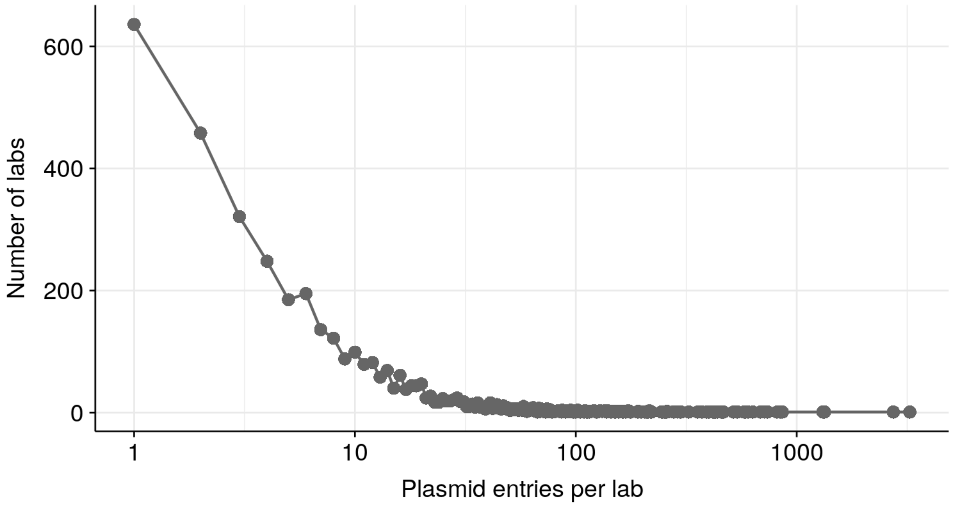

Supplementary Figure 21: Distribution of plasmid entries per lab in the raw Addgene dataset. Number of labs with a given number of sequence entries in the Addgene plasmid database, prior to processing and splitting by Alley et al.[2].

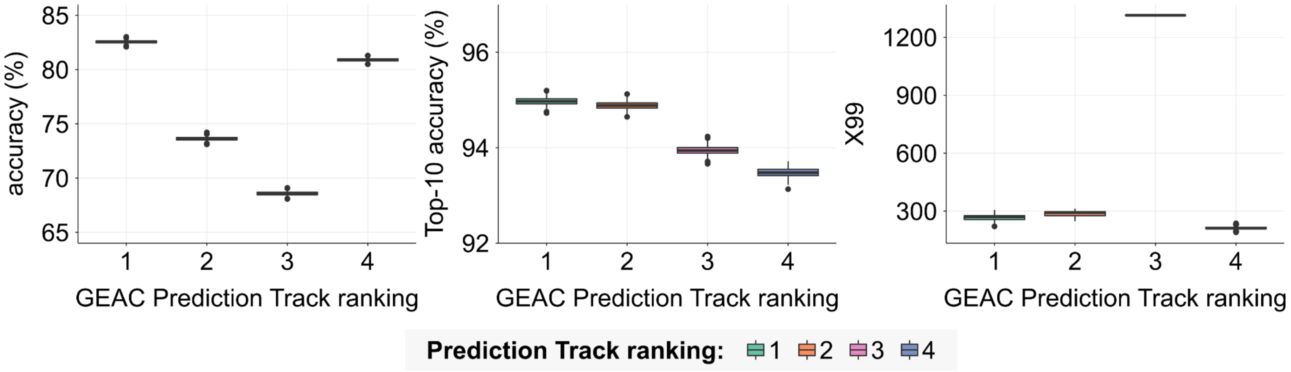

Supplementary Figure 22: Robustness of attribution metrics. The holdout test set was resampled 1000 times, with each subsample comprising 80% of the test set data points sampled without replacement. Attribution metrics ((a) top-1 accuracy, (b) top-10 accuracy, (c) X99) were computed for each sample for each of the four Prediction Track prizewinning teams. Pairwise comparisons between the distribution of scores for each team were made using the KS-test; all pairwise comparisons were significantly different at $p < 0.01$ for all metrics.

3. Supplementary Note: Innovation Track problem description

What follows is the text of the Innovation Track problem description given to participants at the start of the Genetic Engineering Attribution Challenge. The original document also included a list of judges, which has been omitted here.

Innovation track

How would your approach perform in the real world?

Introduction

In the Innovation Track, we challenge you to bridge the gap between your work in the Prediction Track and real-world attribution problems in academia, industry, and forensics. To succeed, submissions should demonstrate how their lab-of-origin prediction models excel in domains beyond raw accuracy. Submissions will be evaluated by a panel of judges, including world leaders in synthetic biology, data science and biosecurity policy. Winners will receive prizes equal to those provided for the Prediction Track.

How to compete – beat the benchmark

Any model that achieves a higher top-10 accuracy score than our BLAST benchmark will qualify for entry to the Innovation Track. Teams that exceed this threshold (i.e. **exceeding 75.6% on the private leaderboard**) will automatically be invited to submit a report for assessment by our panel of judges (see "Assessment", below).

If a large number of teams successfully beat the benchmark and make submissions, altLabs and our partners will perform pre-screening, based on the criteria below, to select which submissions to pass to our judges.

How to win

To succeed in the Innovation Track, you will need to convince experts from a variety of fields that your submission represents valuable progress in solving real-world attribution problems. To do this, you will need to demonstrate – in **plain language** – that your approach is impressive beyond raw lab-of-origin accuracy. This task is fully open-ended: you can write about whatever aspects of your model you think will most impress the judges. Some examples of important criteria you might want to address include:

- **Calibration**: Does your model accurately report its uncertainty about a sample's lab-of-origin?
- **Robustness**: Is your model robust to outliers, changes in the dataset, and unseen labs?
- **Interpretability & biological groundedness**: Explain how your model makes its predictions in a way that would be useful to a biologist or forensic scientist. Do the features it considers important make biological sense? How might you go about integrating the outputs of your model with other biological data sources?
- **Responsible development**: Does your approach thoughtfully address the practical and societal challenges which might arise in real-world applications? Have your design decisions been made

with diverse stakeholders in mind? How might you continue the responsible development of your approach in the future?

This list is not exhaustive; we expect a wide variety of approaches to do well in the Innovation Track. The key is to demonstrate that your model brings us closer to concrete application.

Note on data use: External data may be used in the Innovation Track for the purposes of showcasing the capabilities of the model post-training. External data may not be used for model training or for any use in the Prediction Track.

If you need more inspiration, we've provided three example scenarios below. We emphasize that these are just examples; you do not need to address these specific scenarios to succeed.

Examples

Example 1: Detecting genetic plagiarism in a multi-source plasmid

The International Genetically Engineered Machine (iGEM) competition is the world's premiere student competition in synthetic biology, in which teams of students work together to design, build, test, and measure systems of their own design using interchangeable biological parts. As part of their competition submissions, iGEM teams must provide detailed information regarding the origin of ideas and components they use. With thousands of submissions to review, detecting and investigating misattributed ideas is becoming a challenge for iGEM.

As part of their submission, one iGEM team presents composite parts of their own design, containing sequences derived from multiple different sources. Some of these parts are from the iGEM parts registry, while others are claimed to be of their own design. Could your model identify which components of each plasmid come from which sources, and thus help assess whether all of the team's work have been correctly attributed?

Example 2: Accidental release

A sewage treatment plant notices a strange, unsettling green glow in the...sludge. They send a sample to a local microbiology lab, where it's classified as a *E. coli*, probably the most common laboratory microbe. While harmless, the bacteria have been genetically engineered to express green fluorescent protein: someone is being sloppy with biowaste disposal.

Authorities in the city turn to you to find out which lab is responsible; however, many of the labs in the city are not in your database. In the event that a precise identification is not possible, could your model provide any partial information that would help human investigators narrow the search?

Example 3: Synthesis Screening

A (fictional) DNA synthesis company has a serious problem with fraud: they offer large discounts to academic institutions, but suspect that many private startups are using academic contacts to exploit this, potentially losing millions of dollars in revenue. Given the immense volume of orders, it is impossible to manually inspect each sequence to verify its purported lab-of-origin. So, the company has turned to attribution models to automate verification.

To be useful and economical in this context, an attribution model must overcome certain challenges: among others, it must be very computationally cheap to run, and perform well given only DNA sequence fragments (no phenotype information). Most importantly, to minimize disruption to legitimate business, it should have a very low false-positive rate, while maintaining an acceptably low rate of false negatives. Could your model fulfill these criteria?

Assessment

At the close of the Prediction Track on October 19th, any team whose accuracy exceeds the BLAST benchmark on the **private leaderboard** will be invited to submit a report on their approach. This report should be at most four pages long, with at most two figures, and should:

- Demonstrate a **novel and creative** approach to genetic engineering attribution;
- Demonstrate what capabilities of their model, other than raw accuracy, would make it useful for solving **realistic attribution problems**;
- Show thoughtful consideration of the **societal impact and use** of attribution models;
- Discuss the **limitations** of the model, and how further work might improve it;
- Be **comprehensible** to both technical and non-technical readers.

Assessment of submissions to the Innovation Track will be conducted by a diverse panel of distinguished judges, including top experts in synthetic biology, data science, and biosecurity policy. Each submission will be assessed by multiple judges. As such, to succeed in the Innovation Track, your report should be **comprehensible to both technical and non-technical readers.** Innovation Track prize-winners will be announced alongside those for the Prediction Track.

FAQ

Who can submit to the Innovation Track? Any team that beats our BLAST benchmark on the Prediction Track can submit a report to the Innovation Track.

What is the exact benchmark value we need to beat? The BLAST benchmark for top-10 accuracy is 78.8% on the public leaderboard and 75.6% on the private leaderboard. To qualify for the Innovation Track, your model should score better than 75.6% on the private leaderboard.

What if we have multiple submissions to the Prediction Track? Each team may make at most one submission to the Innovation Track. If your team makes multiple submissions, you must select one to discuss in your Innovation Track entry.

Nobody on our team is a biologist! Can we compete? Any team that beats the BLAST benchmark in the Prediction Track is welcome to compete in the Innovation Track. Collaborating with biology or policy specialists may be helpful for this track, but is not required.

How long will we have to prepare our report? Following closure of the Prediction Track on October 19th 2020, notified participants will have up to two weeks to prepare their submissions to the Innovation Track. However, teams that are confident they've beaten the benchmark are strongly encouraged to prepare their

submissions well in advance. The submission window for the Innovation Track will close at 11:59pm (UTC) on November 1st, 2020.

What should I submit? Participants in the Innovation Track should submit the following:

- The code for your lab-of-origin prediction model. This should be the same model you used in your submission to the Prediction Track.
- Your report, which should explain in plain language how your model fulfills the criteria outlined above.
- Code and data (see below) for generating any figures you include in your report.

The code and data files do not count towards the four-page limit for the report.

How should the report be formatted? Reports submitted to the Innovation Track should be written in English, in PDF format, at most four pages long (Letter or A4), and with at most two figures. There is no minimum length requirement. **To enable blind assessment of submissions, your report should not include the names of your team or team members.** There are no other detailed formatting requirements, but the report should conform to the norms of a good scientific paper: intellectual contributions from outside your team should be acknowledged and cited, and methods should be reported transparently.

What data can I use for the model I submit to the Innovation Track? The model you submit to the Innovation Track should be the same as the corresponding submission to the Prediction Track. As such, **the model should be trained only on the dataset provided for this competition**. External data may also be used in the Innovation Track for the purposes of showcasing the capabilities of the model post-training. As with the Prediction Track, finalists for the Innovation Track will have their model performance validated against an out-of-sample verification set; teams judged to have violated rules regarding data usage will be disqualified.

What data can I use for the figures in my Innovation Track report? While the model submitted to the Innovation Track should be trained only on the data provided for the Prediction Track, you are welcome to use other data to illustrate the capabilities of that model in your Innovation Track report. For example, if your team submits a report based on Example 1 above, you would be welcome to acquire or design some multi-source plasmids to demonstrate your model's ability to distinguish subsequences from different sources. Any additional data you use in this way must be included in your submission to the Innovation Track, alongside the code for your model and figures.

Who owns the intellectual property for my Innovation Track submission? What happens to my model after the competition? As with the Prediction Track, the IP for all prize-winning submissions will be assigned to altLabs upon receipt of prize money. After the competition, altLabs will seek input from various stakeholders – including prizewinning teams – on how best to use these results to promote responsible innovation. altLabs is a non-profit organisation, and will never sell or otherwise monetize prize-winning submissions. The IP for submissions that do not win prizes remains with their respective teams.
